# Structural plasticity of the hippocampus in response to estrogens in female rodents

**DOI:** 10.1186/s13041-019-0442-7

**Published:** 2019-03-18

**Authors:** Paul A. S. Sheppard, Elena Choleris, Liisa A. M. Galea

**Affiliations:** 1 Department of Psychology, Graduate Program in Neuroscience, Djavad Mowafaghian Centre for Brain Health, University of British Columbia, Vancouver, BC V6T 1Z3 Canada; 2 Department of Psychology & Neuroscience Program, University of Guelph, Guelph, ON N1G 2W1 Canada

**Keywords:** Neurogenesis, dendritic spines, sex differences, memory, depression, stress, aging, pregnancy, parity

## Abstract

It is well established that estrogens affect neuroplasticity in a number of brain regions. In particular, estrogens modulate and mediate spine and synapse formation as well as neurogenesis in the hippocampal formation. In this review, we discuss current research exploring the effects of estrogens on dendritic spine plasticity and neurogenesis with a focus on the modulating factors of sex, age, and pregnancy. Hormone levels, including those of estrogens, fluctuate widely across the lifespan from early life to puberty, through adulthood and into old age, as well as with pregnancy and parturition. Dendritic spine formation and modulation are altered both by rapid (likely non-genomic) and classical (genomic) actions of estrogens and have been suggested to play a role in the effects of estrogens on learning and memory. Neurogenesis in the hippocampus is influenced by age, the estrous cycle, pregnancy, and parity in female rodents. Furthermore, sex differences exist in hippocampal cellular and molecular responses to estrogens and are briefly discussed throughout. Understanding how structural plasticity in the hippocampus is affected by estrogens and how these effects can influence function and be influenced by other factors, such as experience and sex, is critical and can inform future treatments in conditions involving the hippocampus.

## Introduction

Numerous investigations in animals and humans have found sex differences in brain and behaviour [1]. While the underlying impetus of these differences is complex and multi-faceted, differences in sex steroid hormones, particularly estrogens, appear to be of clinical and experimental importance [2]. The manifestation, occurrence, and/or severity of brain disorders such as Alzheimer’s disease (AD), autism spectrum disorders, depression, and schizophrenia show differences between the sexes [3–7]. Furthermore, many of these same disorders show sex differences in severity of hippocampus-dependent cognitive symptoms, with greater severity in females with AD [6] and depression [8–11], but greater severity in males with schizophrenia [7]. Cognitive disturbances are likely influenced by disruption to synaptic plasticity in the hippocampus and other regions. These differences are influenced, in part, by estrogens in women, but less is known on whether estrogens may impact these disorders in men [12–14]. In middle-aged and aged women, treatment with exogenous 17β-estradiol – the most bioactive, abundant, and widely expressed of the circulating estrogens in most mammals [15] – may reduce the risk of AD [16], lessen the severity of symptoms of depression [17] or schizophrenia [18], and improve cognition in postmenopausal women [19, 20]. Understanding how estrogens can influence the brain and behaviour is crucial to the development of sex-targeted treatments for brain diseases.

The hippocampus is a brain structure with profound structural and functional plasticity evident across the lifespan in humans and rodents. The integrity of the hippocampus is implicated in learning and memory, anxiety, and stress regulation (e.g. [21, 22]). Furthermore, the hippocampus is implicated in disease states that result in cognitive dysfunction and synaptic function that exhibit sex differences (e.g. autism [5, 23]), schizophrenia [24], depression [25–27], suggesting studying the influence of hormones on hippocampus may inform development of targeted and sex-specific treatments and therapies [5, 28–30].

**Fig. 1:**
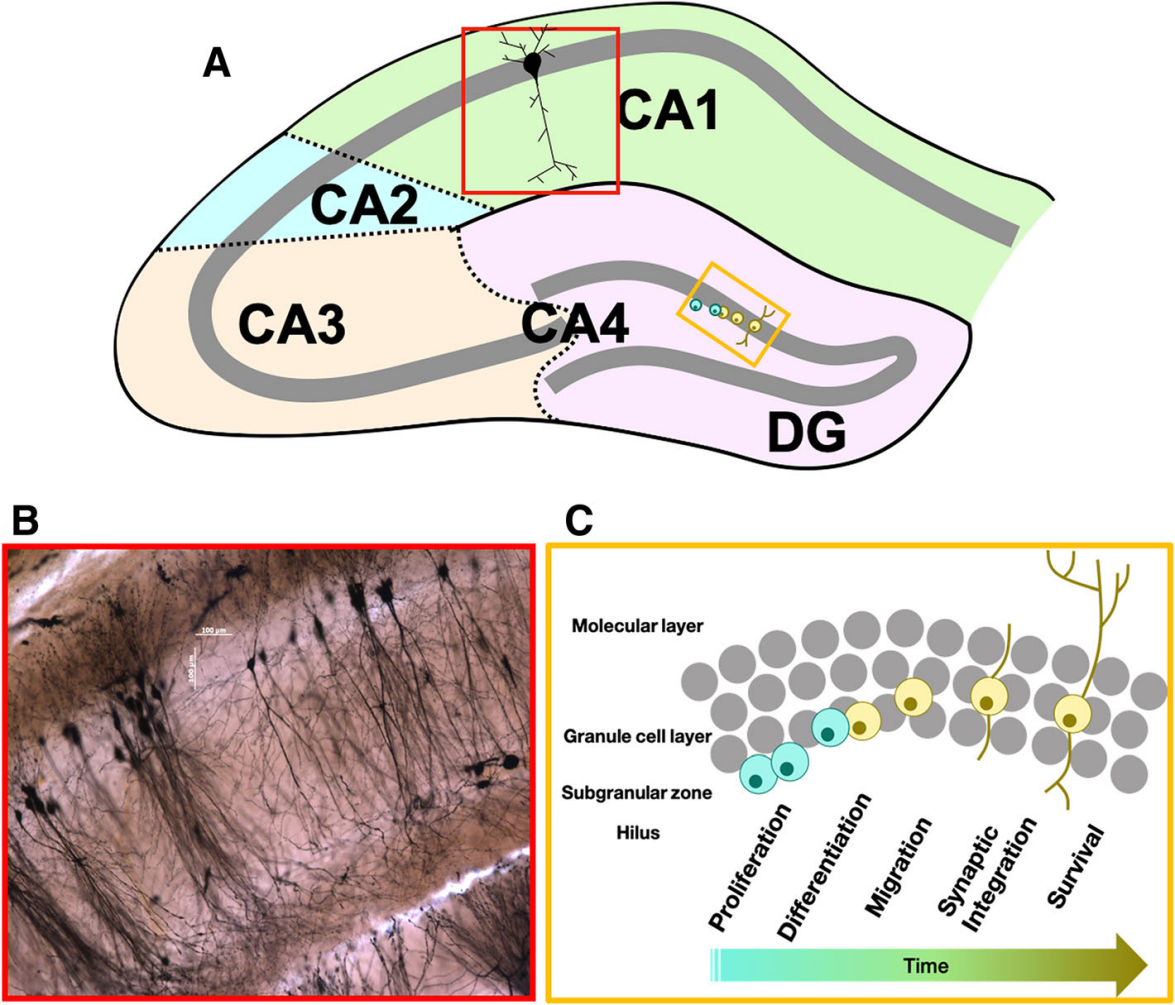
Hippocampal regions, strata, and neurogenesis. **a** Diagram of the major divisions of the hippocampus. Red box shows region depicted in B. Yellow box shows region depicted in C. **b** Image of Golgi-Cox stained hippocampal CA1 neurons from OVX female mouse, captured using 10x objective. Stratum oriens ~40-60% the length of basal dendrite. Stratum radiatum ~30-50% the length of the apical dendrite. Lacunosum-moleculare ~80-100% the length of the apical dendrite. **c** Diagram depicting the stages of adult neurogenesis in the dentate gyrus. DG, dentate gyrus; SO, stratum oriens; SR, stratum radiatum; LM, (stratum) lacunosum-moleculare

Within the hippocampus, changes in dendritic spine number, length, type, and shape may affect neurotransmission and, subsequently, behaviour through modulation of synapses in predominantly glutamatergic neurons. Neurogenesis – the process of proliferation, migration, differentiation, and survival to maturity of novel neurons – allows for new neurons to be integrated into hippocampal networks and modify hippocampal function [31] (Fig. 1c). Both dendritic spine changes [32–34] and neurogenesis [31, 35] exhibit sex differences, respond to estrogens, and allow the hippocampus to maintain plasticity throughout adulthood. Understanding how sex and/or estrogens can affect structural plasticity of the hippocampus is crucial to understanding the functional outcomes of these modifications and to potential future treatments for disorders with hippocampal dysfunction (such as AD, depression, and schizophrenia). Beyond the differences between the sexes, circulating levels of estrogens in females vary by age and reproductive status [36], during pregnancy and parturition [37], and long after reproductive experience [38]. As a result, effects of exogenous estrogens (e.g. from hormone therapies) may also be modulated by these factors but are not always taken into account in the literature.

### Estrogens and estrogen receptors: A short primer

Estrogens can act on estrogen receptors (ERs) within the region to elicit changes in structure and function. ERα, ERβ, and the G protein-coupled estrogen receptor 1 (GPER1) are found in multiple areas of the brain, including the hippocampus, and are expressed in varying densities in both sexes [15, 39–41]. All three receptors are found in the dorsal and ventral horns of the hippocampus and within the CA1, CA2, CA3, and dentate gyrus, and are located in the nucleus and at extranuclear sites such as the dendrites [39, 40]. It should be noted that estrogens can be secreted from the ovaries in females and from adipose tissue in both sexes [42], whereas androgens are secreted from testes in males and from adipose tissue and adrenal cortex in both sexes [43]. However, it is also important to note that estrogens can be synthesized *de novo* from cholesterol in both sexes in the brain or be converted from testosterone *via* aromatase [42].

The majority of neuroendocrine research has explored the effects of 17β-estradiol because it is the most bioactive of the endogenous estrogens in pre-menopausal women [44]. To the best of our knowledge, contributions of estrone and estriol to the formation and modulation of dendritic spines have not been investigated, though these estrogens may be neuroprotective in certain disease and neurological states (e.g. [45–49]). While estrone and estriol are less bioactive than estradiol, their effects on dendritic spines require future investigation, especially with regards to pregnancy (when circulating estriol levels increase greatly as a result of placental production [50]) and post-menopause (when 17β-estradiol levels decline more so than estrone levels leading to estrone becoming the most abundant of the estrogens [51]), periods in which hippocampal dendritic spine numbers are increased [52, 53] and decreased (typically investigated *via* ovariectomy) [32, 54–56], respectively. It should further be mentioned that this review does not cover the effects of phytoestrogens (weak estrogens found in plants) or endocrine disruptors such as bisphenol-A on dendritic spine density or neurogenesis; however, there is emerging evidence that phytoestrogens may affect these types of hippocampal plasticity [57–63].

In this review, we will discuss how estrogens can affect structural plasticity of the hippocampus, dendritic spine morphology and neurogenesis, with the mediating and modulating factors of sex, age, parity, and pregnancy in females.

### Estrogens and dendritic spines

Dendritic spines are small, membranous protrusions from the dendrites of neurons. These structures express many different receptors on their surface and serve as the primary recipients of excitatory synaptic input in the mammalian central nervous system as 90% of excitatory synapses occur on dendritic spines [64, 65]. The plasticity of dendritic spines has been suggested to play a role in motivation, memory, and learning [32, 66]; particularly, the growth of novel spines and morphological changes of pre-existing spines can mediate long-term memory formation [67]. Regional differences exist in dendritic spine density (i.e. the number of spines per unit length of dendrite), from highly spiny regions such as the hippocampus and cortex to spine sparse regions such as the hypothalamus [32]. Within the hippocampus, spine changes in response to effectors (activity, drugs, surgery, compounds, etc.) can vary dependent on region of the hippocampus (e.g. dentate gyrus, CA3, CA1). For instance, orchidectomy in male rats increases dendritic arborization (the degree of branching of the dendrites) in CA3 pyramidal neurons with no effect on the CA1 dendritic arbor [68]. These subregional differences can also be sex specific. For example, an acute stressor in male rats increased apical CA1 dendritic spine density but decreased it in proestrous females [69]. Thus, it is important to keep in mind that there are likely to be regional and sex differences in response to factors such as sex hormones.

Dendritic spines fall into subtypes based predominantly on shape, from stubby, mushroom-shaped mature spines to long, thin, immature spines lacking any sort of synaptic terminal enlargement [70, 71]. Although perhaps too simplistic, it has been suggested that thin spines are the “learning” spines while mushroom spines are the “memory” spines [72, 73]. In this model, thin, learning spines are amenable to experience-induced modifications that lead to the formation of new memories. These experience-induced modifications drive the maturation of thin spines into the more stable mushroom and stubby spines that hold synapses. Unfortunately, many studies investigate the density of dendritic spines but fail to report on spine shape (e.g. [74–78]). There is some debate on the functional significance of these two measures, that is, whether spine number is as important to behaviour as the maturation of spines. It is also hotly contested and debated what the best method of visualization and categorization of spines should entail [72, 73].

It has been known for decades that changes in circulating estrogens alter dendritic spine density in various brain regions in female rodents [52, 79–83], including the hippocampus [54, 55] (Table 1). Across the 4-5 day estrous cycle of the rat, apical CA1 dendritic spine density fluctuates by approximately 30%, with the highest densities coinciding with phases of high circulating estrogens [32, 55]. Ovariectomy (OVX) decreases the density of apical CA1 dendritic spines [54]. This can be reversed by treatment with exogenous estradiol benzoate (EB) [84]. The effect of estradiol, both EB and 17β-estradiol (which metabolizes more rapidly than EB [85]), to increase dendritic spines in apical dendrites of CA1 pyramidal neurons was observed within 24 hours, peaked at 2-3 days, and then gradually declined over the next 7 days [85]. Progesterone treatment, given subsequent to estradiol, served to rapidly increase spine density between 2-6 hours following treatment in OVX rats [85]. Dendritic spine density fell rapidly to baseline levels thereafter [85]. These findings indicate that ovarian hormones can modulate dendritic spine density. Intriguingly, there are reported differences in hippocampal volume across the menstrual cycle in women, with menses phase associated with lower volume than the preovulatory surge [86–88]; but see [89] an effect that is echoed across the estrous cycle in mice [90]. It is tempting to speculate that changes in spines may contribute to these alterations in volume but more studies need to be conducted. Of interest, functional activation patterns are also altered across the estrous cycle in rodents [91], and with menstrual cycle [88, 92, 93] and exogenous estradiol [94] in women, suggesting that ovarian hormones modulate brain activity. Altered activation and volume changes across the menstrual cycle in women could be caused by the modulation of spine and synapse dynamics, dendritic architecture, and/or neurogenesis in the hippocampus.

**Table 1:** Summary of the effects of estrogens on hippocampal dendritic spine density

Reference	Model	Results
Woolley et al., 1990	Intact female rats	CA1 dendritic spine density fluctuates by approx. 30% across estrous cycle; highest densities when circulating estrogens are highest [50]
Gould et al., 1990	Intact and OVX female rats	OVX decreases CA1 dendritic spine density [49]
Woolley & McEwen, 1992	OVX female rats	2 subcutaneous (s.c.) injections of EB (given 24h apart) reverse CA1 dendritic spine density decreases from OVX within 48h following second injection [51]
Woolley & McEwen, 1993	OVX female rats	S.c. EB or 17β-estradiol increases CA1 pyramidal neuron apical dendritic spine density within 24h, peaked at 2-3d, declines over 7d; s.c. progesterone following 17β-estradiol further increased spine density for 2-6h but then levels fell quickly to baseline [52]
Leranth et al., 2003	Intact and GDX male rats	GDX reduces CA1 spine synapse density; s.c. testosterone proprionate increases CA1 spine synapse density in intact males after 2d of treatment; s.c. dihydrotestosterone but not 17β-estradiol increased CA1 spine synapse density in GDX males after 2d of treatment [55]
MacLusky et al., 2004	Intact male rats	Increases in CA1 dendritic spine density driven by s.c. testosterone are not *via* aromatization of testosterone to estrogens [56]
MacLusky et al., 2005	OVX female rats	S.c. 17β-estradiol increases CA1 spine synapse density within 30min and 4.5h; s.c. 17α-estradiol increases CA1 spine synapse density within 30min [63]
Tsurugizawa et al., 2005	*Ex vivo* hippocampal slices from male rats	2h bath in 17β-estradiol or ERα agonist decreased CA3 dendritic excrescence thorns [95]
Kinsley et al., 2006	Intact, pregnant, lactating, and OVX female rats	Pregnant and lactating rats had greater CA1 dendritic spine density than nulliparous intact rats; nulliparous proestrus intact rats had greater CA1 dendritic spine density than nulliparous diestrus or estrus intact rats; OVX rats given hormonal treatment to mimic pregnancy (17β-estradiol and progesterone *via* Silastic implant) had greater CA1 dendritic spine density than OVX controls [135]
Murakami et al., 2006	*Ex vivo* hippocampal slices from male rats	Increased CA1 stratum oriens or lacunosum-moleculare dendritic spine density following 2h bath in 17β-estradiol or ERα agonist [64]
Wallace et al., 2006	Intact and OVX female rats	7wks post-surgery, OVX rats had decreased CA1, but not CA3, dendritic spines compared to intact rats [136]
Mukai et al., 2007	*Ex vivo* hippocampal slices from male rats	Increased CA1 stratum radiatum dendritic spine density following 2h bath in 17β-estradiol or ERα agonist [65]
Phan et al., 2011	OVX female mice	S.c. ERα agonist increased CA1 spine density within 40min; s.c. ERβ agonist decreased spine density and increased spine length within 40min [38]
González-Burgos et al., 2012	Intact male rats	Injection of tamoxifen or raloxifene (route not specified) increased CA1 dendritic spine density; tamoxifen increased thin- and stubby-type spines whereas raloxifene increased thin-, stubby-, and wide-type spines [93]
Phan et al., 2012	OVX female mice	S.c. 17β-estradiol increased CA1 dendritic spine length within 40min [39]
Velázquez-Zamora et al., 2012	OVX female rats	Twice daily treatment (s.c.) with EB increased CA1 dendritic spine density at 3d, but not 10d [125]
Gabor et al., 2015	OVX female mice	S.c. GPER1 agonist increased CA1 dendritic spine density within 40min [41]
Phan et al., 2015	*Ex vivo* hippocampal slices from female mice	17β-estradiol or ERα agonist increased CA1 dendritic spine density within 30min of bath application [40]
Jacome et al., 2016	GDX male rats	Acute s.c. injection of 17β-estradiol or T increased CA1, but not DG, dendritic spine density 30min or 2h following treatment [59]
Tuscher et al., 2016	OVX female mice	Intrahippocampal 17β-estradiol increased CA1 basal and apical dendritic spine density within 30min or 2h of treatment; intracerebroventricular 17β-estradiol increased CA1 basal and apical dendritic spine density within 2h via ERK and mTOR pathways [42]
Mendell et al., 2017	Intact and OVX female rats, intact and GDX male rats	Proestrus intact females had greater CA1 apical dendritic spine densities than metestrus intact or OVX females; proestrus intact females had greater CA3 apical dendritic spine densities than OVX females; GDX males had increased CA3 dendritic branching than intact males; OVX had minimal effects on dendritic branching [32]

OVX, ovariectomized; GDX, gonadectomized; EB, estradiol benzoate; ER, estrogen receptor; GPER1, G-protein-coupled estrogen receptor 1; s.c., subcutaneous

In addition to the influence of estrogens in females, dendritic spines in the CA1 region also fluctuate in males with hormones. Interestingly, long-term changes in dendritic spine density in this region is driven by testosterone [95], but not by aromatization of testosterone to estradiol [96]. Similarly, androgens, but not estrogens, also upregulate neurogenesis in the dentate gyrus of adult males [97, 98], as reviewed in a latter section (*Estrogens and neurogenesis*). Gonadectomized male rats have reduced CA1 dendritic spine density when compared with intact males, which is recovered by testosterone propionate or dihydrotestosterone treatment, but not 17β-estradiol, for 2 days [95, 99]. Testosterone or dihydrotestosterone rapidly (within 2h) increased in CA1 pyramidal neuron dendritic spine density in *ex vivo* hippocampal slices from adult male rats [100]. Similarly, in *ex vivo* hippocampal slices from adult male rats, a 2h bath in 17β-estradiol (1nM) increased dendritic spine density in the strata oriens, radiatum, and lacunosum-moleculare of the CA1 [101, 102]. In male rats, both testosterone and 17β-estradiol *in vivo* also rapidly increased dendritic spine density in the CA1 region, but not in the dentate gyrus, 30 minutes or 2 hours following treatment [103]. Collectively, these findings suggest that estrogens increase dendritic spines in males only *via* rapid mechanisms of action, whereas androgens increase spines both rapidly and long-term. Differences between long-term and rapid effects of steroid hormones, especially estrogens, have been of increasing interest in recent years [33, 34, 104–106].

Acute, exogenous 17β-estradiol or EB substantially (i.e. as much as 50%) increases dendritic spine and synapse density in both rapid and longer timeframes in the CA1 region of the hippocampus of female rodents [74–78, 84, 101–103, 107]. Woolley and McEwen [84] initially found that losses in dendritic spine density following OVX in female rats can be overcome through two 10μg subcutaneous injections of estradiol benzoate (EB; given 24h apart) when evaluated 48h following the second injection. More rapid effects of estrogens have since been observed. Within 4.5h of 17β-estradiol treatment in OVX female rats (45μg/kg; a dose previously found to enhance visual and place memory [108]), spine synapse density in the CA1 was significantly increased [107]. Within 30 minutes, either 17β-estradiol (45 or 60μg/kg) or 17α-estradiol (15 or 45μg/kg) increased spine synapse density in the same model [107]. In OVX female mice, a single subcutaneous injection of 17β-estradiol (1.5, 2, or 3μg/kg) increased dendritic spine density in CA1 stratum radiatum, but not lacunosum-moleculare within 40 minutes [75] (see Fig. 1 for hippocampal subregions). Treatment of 17β-estradiol directly into the hippocampus of female mice (30 minutes or 2h following 5μg/hemisphere) [78] or to *ex vivo* hippocampal slices from female mice (50nM for 20-30 minutes, equivalent to the intrahippocampal dose that facilitated social recognition, object recognition, and object placement in a separate set of mice in the same study [6.81pg/hemisphere]) [76] rapidly increased CA1 dendritic spine density. It is important to note here that the contribution of these novel spines to behaviour is unknown. Experiments utilizing *in vivo* estrogen administration almost exclusively (one exception is [109]) used behaviourally naïve animals. Whether these spines are utilized in behaviour or are modulated by behaviour remains, as of yet, to be determined. While estrogens affect a number of behaviours [105, 110, 111], the next section will focus on their effects on hippocampus-dependent learning and memory.

### Estrogens in the hippocampus: Learning and memory

**Fig. 2:**
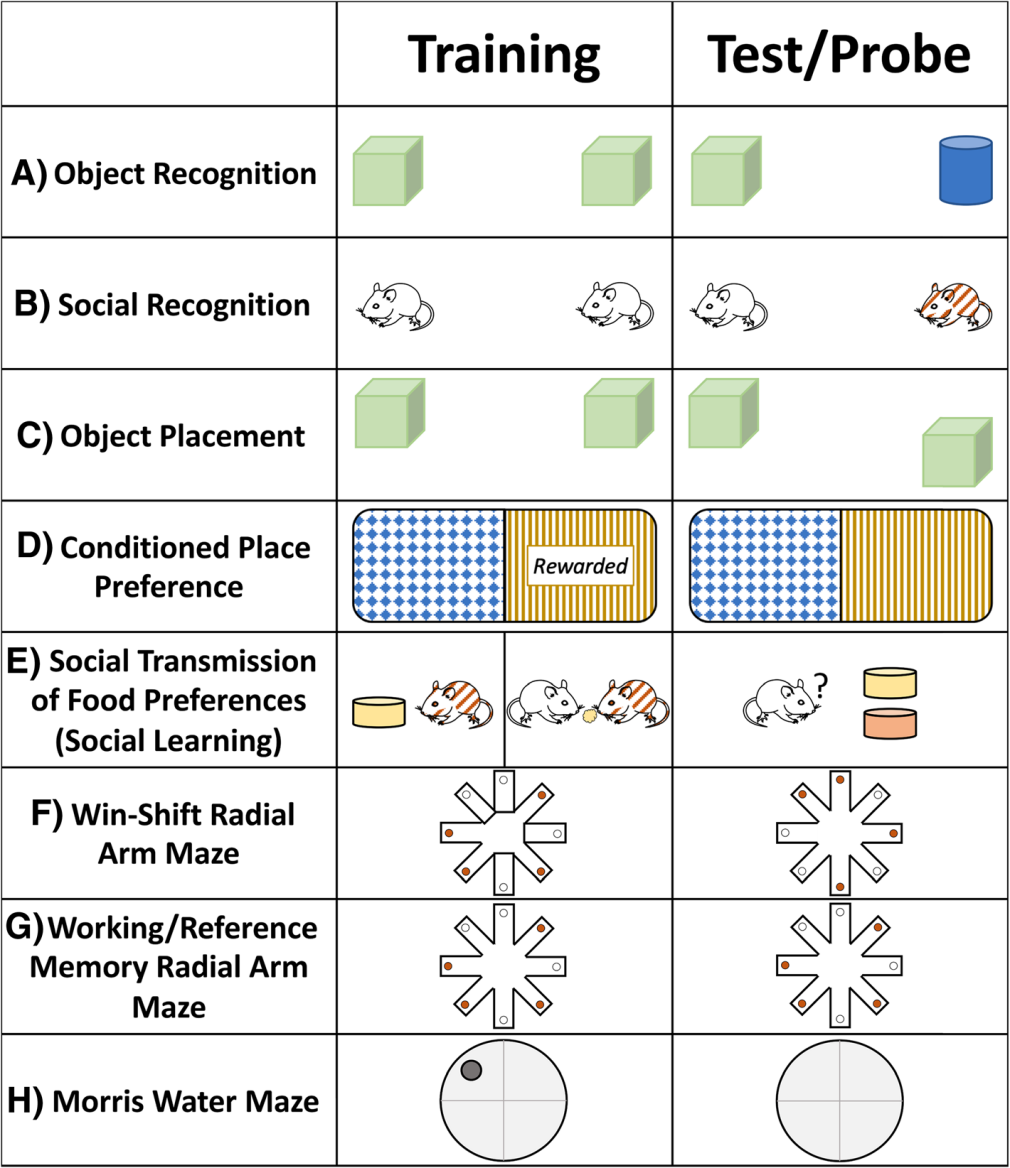
Overview of behavioural tasks affected by estrogens and mentioned in this review. **a** Object recognition, **b**) social recognition, and **c**) object placement tasks take advantage of rodents’ innate preference for novelty. In each of task, a test rodent is presented with stimuli (typically two) to explore during training. Upon test, one stimulus is replaced with a novel stimulus (object/social recognition) or moved to a novel location. **d** In conditioned place preference, an animal is rewarded in one of two distinguishable contexts. A probe trial then explores the amount of time spent in the two contexts. **e** In the social transmission of food preferences, a “demonstrator” animal consumes a novel flavoured diet. They are then paired with an “observer” for an interaction period in which the observer will smell the novel flavoured diet on the demonstrator’s breath. When given a choice between the flavoured diet smelled on the demonstrator’s breath and another novel diet (both diets are novel to the observer), an animal with intact social learning will prefer the demonstrator’s diet. **f** In the win-shift version of the radial arm maze, rodents are placed in the maze and allowed to enter only a subset of the arms in order to receive rewards. Upon test phases, all arms are open, but rodents are only rewarded at the termini of formerly un-baited arms. Entries into previously baited arms are reference memory errors, whereas re-entry into arms entered during the test phase are working memory errors. Similarly, **g**) in the working/reference memory radial arm maze task, rodents are repeatedly rewarded in the same arms. Entries into never-baited arms are reference memory errors, whereas re-entries are working memory errors. **h** In the Morris water maze, an animal learns to swim to a hidden platform to escape. Probe trials then evaluate the amount of time the animal spends swimming in the quadrant previously containing the platform

While a causal link between the physiological (e.g. increase in dendritic spine density) and behavioural (e.g. facilitation of short-term social recognition memory) has yet to be proven, many of these effects occur within similar timeframes. Estrogens rapidly increase synapse density both *in vivo* [107] and *in vitro* [112, 113], suggesting that pre-synaptic input (i.e. synaptic transmission to estrogen-treated neurons) may also be involved in the 17β-estradiol mediated changes (impairments or enhancements) in learning and memory that have been observed within the same rapid timeframe (e.g. [75, 76, 108, 114–122]). Learning and memory (see [123] for disambiguation of these terms) are not a singular process but are comprised of several steps including encoding, storage, and retrieval of information [72]. In brief, typically, learning is considered to be the acquisition or encoding of information to memory, whereas memory could be considered the storage and retrieval of information [123]. Memory can be divided into many different subtypes (e.g. episodic and semantic memory, declarative and non-declarative memory) and there are many reviews written on the subject (e.g. [124, 125]). One way we find it useful to categorize memory is working versus reference memory. Reference memory is considered long-term memory for events or stimuli that stay stable over time [126], whereas working memory can be defined as trial-unique information to guide prospective action [127, 128]. Reference memory relies more on the dorsal hippocampus [129, 130] while working memory relies more on the ventral hippocampus (and prefrontal cortex) [129–132]. Different tasks that rely on the integrity of the hippocampus can influence reference and working memory to different degrees and, not surprisingly, estrogens can have different influences on these different types of learning and memory. We illustrate a number of tasks mentioned in this review that are used to assess different forms of learning and memory in Fig. 2. It is important to note that there are different versions of the tasks shown in Fig. 2 and, thus, the descriptions are not exhaustive. A thorough discussion of different forms of memory and how the hippocampus may be involved is beyond the scope of this review, but we direct the readers to excellent reviews on this subject [133–136].

Crucially, 17β-estradiol given during consolidation/storage can improve memory of a reference memory task of object recognition or object placement memory (tasks that can involve the dorsal hippocampus) 24-48 h after 17β-estradiol exposure [115–117, 119, 122]. Other studies examining performance in the spatial working reference/memory version or working memory win-shift version of the radial arm maze (hippocampus-dependent tasks) show facilitated acquisition at lower doses of EB or impaired acquisition at higher doses of EB [118, 137]. Performance in other spatial memory tasks show a similar dose response in performance with 17β-estradiol in both humans [138] and rodents [139–141]. In addition, systemic or intrahippocampal administration of 17β-estradiol 15 minutes prior to training in an object, social, or place recognition task facilitated performance when tested 40 minutes later in OVX female mice [74–76]. It is clear that 17β-estradiol can facilitate or impair various aspects of working and reference memory dependent on dose, course of treatment, when during encoding, consolidation, and/or retrieval 17β-estradiol is given, and the type of task (e.g. what brain areas are recruited during the task). Task performance may be influenced by 17β-estradiol’s effects on synaptic plasticity, including influence on dendritic spines, the putative structural and integral compartments of learning/memory within the synapse.

### Estrogens and “two-step wiring plasticity”

**Fig. 3:**
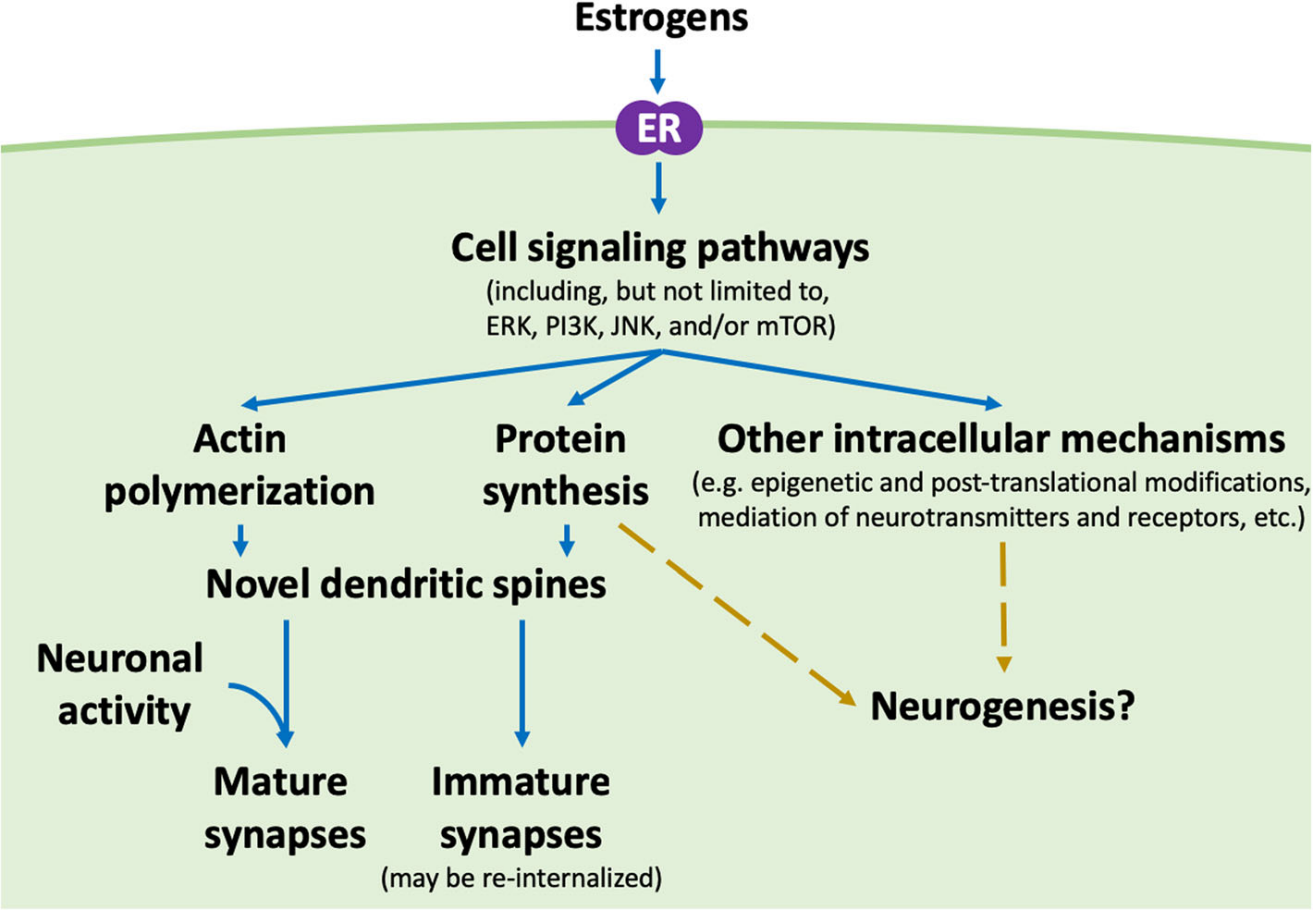
Suggested non-genomic, intracellular mechanisms driving dendritic spine changes and neurogenesis by estrogens. We hypothesize that estrogens bind to estrogen receptors (membrane bound or intracellular) which go on to activate cell signalling pathways, including, but not limited to the ERK, PI3K, JNK, and/or mTOR pathways. Cross-talk between these pathways is common. These have downstream effects on a number of intracellular mechanisms, including protein synthesis and actin polymerization. Through actin polymerization and protein synthesis, novel spines or “silent” synapses are created, which can become mature synapses following neuronal activity. If unused, the novel spines do not mature and are instead re-internalized. Other intracellular mechanisms, such as epigenetic or post-translational protein modifications and mediation of neurotransmitters and/or receptors, are likely also involved. The contributions of cell signalling pathways and other intracellular mechanisms in the effects of estrogens on neurogenesis remain to be explored

The combination of 17β-estradiol increasing spines and enhancing memory suggested the idea of “two-step wiring plasticity” (also known as “sample and hold” theory [142]; see [66] for a thorough explanation). Briefly, in Step 1, acute application of 17β-estradiol to cultured cortical neurons from embryonic day 18 rats (mixed sex [personal communications]) led to a rapid, transient increase in the density of dendritic spines in an extracellular signal-regulated protein kinase (ERK)-dependent manner, along with the presence of silent synapses (i.e. synapses where the postsynaptic membrane contains few alpha-amino-3-hydroxy-5-methyl-4-isoxazole-propionic acid (AMPA)-type glutamate receptors) [112]. Following this, in Step 2, when N-methyl-D-aspartate (NMDA) receptors were activated, the increases in dendritic spine density and silent synapse number persisted up to 24 hours [112]. NMDA receptor activation was necessary for dendritic spine increases to persist. While this effect was investigated in cultured embryonic cortical neurons, rapid “two-step wiring plasticity” may be a mechanism by which estrogens can exert their enhancing effects on learning in other brain regions in adults (e.g. [114]). That is, estrogens may affect learning by priming neurons to form lasting connections by first creating silent synapses and increasing dendritic spine density (Step 1) – likely through actin cytoskeleton dynamics [66] and *de novo* protein synthesis [106, 143–145] – followed by stimulation (Step 2), leading these neurons to undergo long-term potentiation (LTP) [146, 147]. In this way, novel synapses are formed selectivel by exposure to estrogens. Only when silent synapses are present and when the neuron is activated (receives stimulation) do those synapses that get utilized in the neuron’s activity develop into their fully functioning forms (Fig. 3). However, other potential explanations exist for the mechanisms behind the effects of estrogens on learning, including those that may or may not involve changes to dendritic spine density (e.g. glutamate receptor shuttling and/or stabilization [148]). Through what mechanisms estrogens affect learning and memory, and whether novel dendritic spines and/or LTP are involved, requires further investigation.

### Estrogens do not always improve memory or LTP

As noted above, estrogens do not always enhance learning and memory. OVX female mice given 17β-estradiol *via* drinking water for 5 weeks improved object recognition memory but impaired spatial reference memory at the middle physiological dose [149]. Similarly, chronic treatment with high doses of EB impaired (whereas low doses facilitated) spatial working memory in radial arm maze [118] and conditioned place-preference [150] in OVX female rats. Further, acute administration of high doses of 17β-estradiol and progesterone can also impair performance in the standard reference memory version of Morris water maze [151]. There is agreement in the human literature as well, with high endogenous levels of 17β-estradiol associated with poorer performance on spatial tasks [138] and cognitive function (assessed using the Montreal Cognitive Assessment scale [152]) and high exogenous 17β-estradiol impairing recognition memory [94]. It is important to acknowledge that estradiol leads to curvilinear influence on hippocampal-dependent performance, with low and high levels impairing but a medium dose improving performance on a variety of tasks.

Estradiol also can have dramatically different effects on LTP as well, dependent on dose and region. For example, proestrus was associated with increased hippocampal LTP in the Schaffer collateral-stratum radiatum (CA1) pathway in adult female rats [153]. A recent study shows that in *ex vivo* hippocampal slices from adult female mice, 17β-estradiol (15 minutes) reduced CA1 miniature excitatory postsynaptic current frequency and firing in response to AMPA [76]. However, when EB is given in longer timelines (2 *in vivo* injections, 24 hours apart), in *ex vivo* hippocampal slices from OVX female rats, excitatory synaptic transmission in the CA1 was potentiated through increases in presynaptic vesicular glutamate release [154]. These findings collectively suggest that 17β-estradiol can transiently decrease excitation in the CA1 but increase excitation over longer timeframes. Differences in timing (rapid [15 min] vs longer-term [48 h]), route of administration (subcutaneous injection, implant, intercranial infusion, *in vitro* bath application, etc.), and experimental model (species, sex, *in vivo, ex vivo, in vitro,* etc.) between studies may underlie these, and other, between-study differences. For instance, in the above electrophysiology experiments, decreased firing resulted from application of estradiol to *ex vivo* hippocampal slices concurrent to measurement [76, 102], whereas *in vivo* treatment with EB 2 days prior to sacrifice and measurement resulted in an increase in synaptic transmission [154]. Here, differences in timing and/or model may underlie the opposing effects. Similarly, the rapid effects of estrogens involve intracellular signalling which may or may not lead to genomic products [33], whereas longer-term actions of estrogens, such as those in studies using chronic hormone treatments, may involve both genomic actions of estrogens (classical and non-classical) and ongoing, non-genomic, rapid effects. It is important to consider these factors as they may make comparing the results of different studies difficult.

There are important considerations when observing the effects of estrogens on LTP and LTD. Finding an appropriate tetanus can be difficult, as hippocampal excitability and seizure threshold increase with high 17β-estradiol. For example, the hippocampus is more prone to seizure during proestrus (high circulating estrogens [153, 155]) or following chronic high-dose 17β-estradiol replacement (dorsal, but not ventral hippocampal seizures [156]). Furthermore, the hippocampus can be hyperexcitable following systemic administration of EB [157] and there are dose-dependent seizure risk associations found in women with epilepsy [158]. Additionally, there has been much discussion about the lack of statistical power in many neuroscience studies [159]. Contradictory findings in similar experiments could be driven by inadequate sample sizes. Researchers are urged to consider statistical power when planning future experiments, especially when sex or hormonal differences are involved, for example, often estrous cycle analyses are underpowered due to subdividing an already small sample size [1, 2]. However, it is equally important is to consider all the variables in the experiment (see [160]), given that tetanus, region of the hippocampus, age, and experience matter for LTP outcomes. As such, all aspects must be considered and the LTP and estrogens story must not be generalized when multiple areas or stimulation paradigms are used. Careful control of experimental parameters (e.g. consistency and control of experimental conditions, such as cue choice in spatial tasks) is adequate to maintain statistical power in neuroscience research [160].

As suggested earlier, the effects of estrogens on behaviour are often dose dependent and follow an inverted U-shape dose response curve with intermediate doses showing the greatest effects [161] (e.g. [76, 77, 94, 114, 149, 162]). Similar dose response curves have been observed in the rapid effects of estrogens on dendritic spines [74, 76]. As such, investigations into the effects of estrogens on either behaviour or cellular morphology should take dose response into consideration. Furthermore, longer term exposure to estrogens can similarly have dose dependent responses on learning and memory, with low levels of 17β-estradiol enhancing spatial working memory and high levels of estradiol impairing spatial working and reference memory [150, 163, 164]. Studies have also shown that, whereas there is dose dependent facilitation in contextual fear conditioning by 17β- and 17α-estradiol, estrone results in dose dependent impairments in contextual fear conditioning [165]. Thus, it is important to keep in mind that the number of injections, dose, and type of estrogen(s) utilized along with the type of memory task investigated and when during acquisition or retrieval estrogens are given, are critical to the learning outcomes.

### Molecular mechanisms of spine changes

The contributions of the different subtypes of ERs to the modulation of dendritic spines are not yet fully understood. Increases in apical and basal pyramidal neuron spine density were found within 2 hours of administration of ERα agonist PPT in the CA1 hippocampus of male rats, while ERβ agonist DPN did not produce such effects [101]. Twenty-four hours following treatment with mixed agonist-antagonist selective estrogen receptor modulators, raloxifene and tamoxifen, male rats had higher CA1 dendritic spine densities than vehicle treated controls [166]. Tamoxifen increased thin- and stubby-type spines over controls and mushroom-type spines over both vehicle and raloxifene treated males, whereas raloxifene treated males showed increases in thin-, stubby-, and wide-type spines over control males [166]. Within 40 minutes of systemic administration, 17β-estradiol [75], PPT [74], or GPER1 agonist G-1 [77] in young adult OVX female mice increased dendritic spine density in the stratum radiatum of the CA1 hippocampus, with PPT also increasing dendritic spine density in the lacunosum-moleculare [74]. In all, ERα and the GPER1 seem to drive the effects of estrogens in this region, whereas other regions show more involvement from ERβ (e.g. the cortex [112] and the medial amygdala [114]).

While the link between changes in dendritic spines and behaviour has not been causally demonstrated in these studies, many of the same doses of these estrogens and ER agonists that increased dendritic spine density also facilitated short-term social recognition, social learning, object recognition, and/or object placement memory in a separate group of OVX female mice in the same timeline (40-45 minutes) as spine changes [33, 34, 74–77, 114, 167]. Conversely, systemic administration of DPN impaired social recognition, facilitated object placement, did not affect object recognition performance in OVX female mice, and decreased dendritic spine density and length in the lacunosum-moleculare [74]. In *ex vivo* hippocampal slices, 17β-estradiol and PPT, but not DPN, rapidly increased spine density in the stratum radiatum and stratum oriens subregions of the CA1 [76]. Female OVX mice administered a memory-improving dose of 17β-estradiol into the dorsal hippocampus had increased basal and apical dendritic spine density in CA1 pyramidal neurons 30 minutes and 2 hours following hormone treatment [78]. Furthermore, a memory-improving dose of intracerebroventricular 17β-estradiol similarly increased basal and apical dendritic spine density in CA1 pyramidal neurons within 2 hours in an ERK- and mammalian target of rapamycin (mTOR)-dependent fashion [78]. Further study is required to conclusively determine the involvement of estrogen-facilitated dendritic spine changes in learning and memory.

The majority of studies have utilized behaviourally-naïve animals when examining spinogenesis. Interestingly, behavioural training (Morris Water Maze) interfered with EB-facilitated increases in CA1 spine density [109]. Similarly, more recent investigations have found a lack of CA1 dendritic spine density increases in animals treated with 17β-estradiol prior to acquisition in a rapid short-term social recognition memory testing (Sheppard & Choleris, unpublished results). One potential explanation is that estradiol treatment is increasing overall spine number, but, upon activation or experience, only a subset of spines, perhaps those involved in the learning, persists. Further investigation into whether novel spines produced following 17β-estradiol treatment remain, mature, or are utilized in behaviour is required.

Intriguingly, in the CA3 region of the hippocampus, there are distinct spines called thorny excrescences that are the postsynaptic synapses from the mossy fiber inputs from granule cells in the dentate gyrus [168]. In hippocampal slices from adult male rats, 1nM 17β-estradiol for 2h decreased CA3 excrescence thorns [169]. This decrease was blocked by AMPAR, but not NMDAR, antagonism and MAPK signalling inhibition [169]. ERα is believed to drive this decrease as PPT, but not DPN produced a similar effect and ERα was present at CA3 mossy fibre terminals [169]. Notably, CA3 dendritic spine density does not vary across the estrous cycle [54, 55]. Ovariectomized female rats had decreased dendritic spine density in proximal, medial, and distal regions of the apical dendrites of pyramidal neurons in the CA1, whereas the CA3 had decreases in proximal and distal regions only, when compared with gonadally intact females in proestrus, a high 17β-estradiol phase of the estrous cycle [68]. Females in metestrus also had reduced spine density in the medial region of the CA1 when compared to those in proestrus [68]. Interestingly, orchidectomy in males significantly increased dendritic branching in the CA3 whereas OVX in females had minimal effects on the dendritic arbor [68]. Although the CA1 is more often studied, there are clearly sex differences in the effects of sex hormones on CA3 dendritic spines and arborization that require further investigation.

Of particular interest in dendritic spine modulation is the actin cytoskeleton [67] – a dynamic network of actin protein filaments and associated actin binding proteins – which is highly plastic and serves structural roles in many cell types [67]. A number of studies have found the actin cytoskeleton to be pivotal in the formation, elimination, motility, stability, size, and shape of dendritic spines (e.g. [170–173]). Estrogens have been found to affect the remodeling of the actin cytoskeleton (Fig. 3) by rapidly stimulating the RhoA/ROCK (RhoA kinase) pathway and activating Rac/p21-activated kinase (PAK) signalling in neurons [174–177]. Furthermore, intrahippocampal administration of latrunculin A, an inhibitor of actin polymerization (the process by which globular actin [G-actin] units combine to form filamentous actin [F-actin]), blocked the rapid GPER1-mediated enhancements of long-term object and spatial memory consolidation in OVX mice [178]. As such, effects on the pathways affecting the actin cytoskeleton dynamics within dendrites are a potential mechanism by which estrogens facilitate learning and memory on a rapid timescale.

In addition to remodeling of the actin cytoskeleton, protein synthesis has been implicated as a critical factor in changes to dendritic spine morphology [143, 179], as well as in learning and memory (e.g. [180–189]). The enlargement and stabilization of dendritic spines requires synthesis of new proteins, and specific subsets of mRNAs are actively transported to and stored in neuronal dendrites in order for local protein synthesis to be synapse- or spine-specific [143]. It has been known since the 1960s that estrogens increase protein synthesis [190, 191]. Through their classical, genomic actions, estrogens, *via* dimerized ERs binding to estrogen response elements on target genes, regulate gene transcription and subsequent protein synthesis [192]. Additionally, recent evidence suggests that local protein synthesis (i.e. the synthesis of novel proteins from mRNA stored in the dendrites, independent of gene transcription) may underlie many of the effects of estrogens on dendritic spines, both long-term and rapidly [34, 193, 194].

In the hippocampus and in cultured hippocampal pyramidal neurons, estradiol (either EB [195–197] or 17β-estradiol [174, 194, 198]) alters synaptic protein expression *in vivo* [195–197] and *in vitro* [174, 194, 197]. In the CA1 dorsal hippocampus of male rats, EB, PPT, and DPN increased post-synaptic density protein 95 (PSD-95) – a post-synaptic scaffolding protein – and AMPA-type glutamate receptor subunit GluR1, with DPN also increasing GluR2 and decreasing GluR3 [195]. Following 2 days of EB treatment in OVX female rats, pre-synaptic markers synaptophysin (vesicular protein) and syntaxin (pre-synaptic membrane-bound protein) and post-synaptic marker spinophilin (spine homeostasis protein) were significantly increased in the CA1 [196]. Interestingly, OVX female rats show an increase in spinophilin 2 days following EB treatment, whereas levels are decreased in gonadectomized males [198]. OVX female rats injected twice daily with 10μg of EB had increased CA1 dendritic spine density at 3 days, but not 10 days, with increases of synaptophysin expression at both timepoints [199]. Similarly, spinophilin was increased in primary hippocampal cultures from embryonic day 18 rat embryos (sex not discussed) 24 hours following EB treatment in a CaMKII-dependent manner [197]. In the CA3 of OVX female rats, synaptophysin was increased in response to high and middle doses of estrone (10 and 1μg, respectively) and low dose 17β-estradiol (0.3μg) in conjunction with contextual fear conditioning, without these increases in synaptophysin correlating with cognition [165].

In a well-established *in vitro* model system of differentiated NG108-15 neurons, Akama & McEwen [200] found that PSD-95 protein, but not mRNA, levels were increased rapidly following 17β-estradiol treatment in an Akt-dependent manner suggesting that, in these cells, 17β-estradiol was eliciting an increase in translation, which was independent of DNA transcription. Enhanced consolidation of longer-term object recognition memory (tested 48h post-training) by intrahippocampal administration of 17β-estradiol may require local synthesis of proteins as mTOR is rapidly activated by ERK and Akt signalling cascades leading to phosphorylation of translation initiation proteins eukaryotic initiation factor 4E-binding protein (4E-BP1) and p70 ribosomal S6 kinase (S6K) and is required for 17β-estradiol-facilitated memory enhancements [117]. Similarly, mTOR is rapidly activated (phosphorylated) in a calpain-dependent manner 15 minutes following acute 17β-estradiol treatment to *ex vivo* hippocampal slices [174]. Expression of activity-regulated cytoskeleton-associated protein (Arc), a protein known to be rapidly translated in response to activity, was also up-regulated in these slices and these effects may depend upon GPER1 signalling and not ERα or β [174]. Collectively, this evidence suggests that increases in spine density and synapse formation in the hippocampus may involve a 17β-estradiol-triggered protein synthesis, often in a dendrite-localized fashion (Fig. 3). However, novel protein synthesis is not required for the rapid 17β-estradiol-facilitated formation of new dendritic spines in cultured cortical neurons (from embryonic day 18 rats; mixed sex [personal communications]) [112]. It is unclear whether protein synthesis, mediated by a mTOR pathway, is required for the maturation and stabilization of novel spines in this region or whether experiment model (*in vitro* vs. *in vivo*) accounts for differences in results [106, 113, 194]. Thus, further investigation into whether protein synthesis is necessary for increases in hippocampal dendritic spine number, either rapidly or over longer timeframes, is warranted (Fig. 3).

### Estrogens and neurogenesis

Estrogens not only modulate dendritic spines but also the birth and survival of new neurons in the dentate gyrus (for review, see [35]). There have been a few reviews specifically on this topic and the reader is directed to these reviews for a more in-depth discussion [31, 35, 201]. Briefly, the process of neurogenesis involves a number of steps leading to mature neurons (Fig. 1c). First, neural progenitor cells that reside in the subgranular zone in the dentate gyrus undergo asymmetrical proliferation. The daughter cells then can differentiate into neurons, glia, or progenitor cell types. If the cell fate is that of a neuronal phenotype, the cell migrates a small distance into the granule cell layer. Eventually, the new neuron will express mature neuronal proteins (~2-3 weeks in rats and ~4 weeks in mice), establish synaptic connections with CA3 neurons (and within the DG), and become electrophysiologically active [202, 203]. Estrogens can influence neurogenesis by acting on any of these processes, resulting in either a net increase or decrease in levels of new mature neurons.

It bears mentioning that there are some studies that question the extent of adult neurogenesis in the hippocampus of humans. A recent paper by Sorrells and colleagues [204] cast doubt on the presence of adult hippocampal neurogenesis in humans, although claims of near-absent neurogenesis in humans [205, 206] and non-human primates [207, 208] have been made before. This was followed shortly thereafter by a paper by Boldrini and colleagues [209] which reached the opposite conclusions. Using multiple methods – bromodeoxyuridine [210], doublecortin [211], and ^14^C dating [212] – numerous studies show evidence for hippocampal neurogenesis in humans, but, as in rodents, there are precipitous declines in levels of neurogenesis with age [213]. Flaws in the design of the Sorrells et al study have been discussed [214], but, briefly, the actual numbers of DCX+PSA-NCAM cells are not given and PSA-NCAM is not exclusive to the dentate gyrus or to new neurons [214]. The majority of studies observe low, but detectable, levels of neurogenesis in adult humans [214]. Other studies have suggested immunohistological methods underestimate the numbers of new neurons in human hippocampus [209, 214] and the data using ^14^C dating suggests much greater levels of neurogenesis exist in the human brain than was previously believed. As our review focuses on experimental animal studies, we direct readers to these papers for discussion on the existence and extent of adult hippocampal neurogenesis [204, 209, 214–217]).

Research indicates that estrogens rapidly upregulate cell proliferation within 30 minutes [218] a time-frame similar to that observed for the rapid increase in dendritic spines. However, with prolonged exposure to EB (48 h), a decrease in cell proliferation is observed [219, 220]. This biphasic effect is most likely due to the homeostatic nature of neurogenesis [221, 222] but also to the ability of 17β-estradiol to upregulate adrenal steroids, as adrenalectomy eliminates the downregulation of cell proliferation in the dentate gyrus 48 h later [223]. ERα and ERβ agonists upregulate cell proliferation in adult female rats [224]; however, the ability of 17β-estradiol to increase cell proliferation is not dependent on GPER1 or NMDAR activation [224–226]. The upregulation of neurogenesis with ovarian hormones is also seen across the estrous cycle, with female rats showing the highest levels of cell proliferation during proestrus [227, 228] (but see [229]). Estrogens do not appear to alter cell fate/differentiation but do influence survival of new neurons, although this latter effect depends upon the type of estrogen, whether estrogens are administered throughout the survival period, and/or whether animals perform a cognitive task [230–232]. In short, 15 days of EB can decrease survival of new neurons independent of the influence on cell proliferation [232]. However, if 17β-estradiol is given right before administration of bromodeoxyuridine – a thymidine analog used to identify proliferating cells – an increase in survival of new neurons is seen in rats that have also undergone water maze training [231]. In contrast, rats given estrone showed a decrease in survival of new neurons [231]. These findings indicate that not all estrogens increase neurogenesis in the hippocampus. Intriguingly, spatial training and age influence how Premarin, a hormone therapy comprised of 50% estrone sulphate and 0.1% estradiol sulphate, increases neurogenesis [230, 233]. Premarin increases survival of new neurons in radial-maze trained rats but not in cage controls [230]. In middle-aged nulliparous or primiparous rats (i.e. rats who have never given birth to a litter and rats who have had one litter, respectively), lower doses of Premarin decreased survival of new neurons in rats that were also trained in the Morris Water Maze [233]. Recent work also suggests that long-term exposure to estradiol, but not DPN or PPT, increases survival of new neurons in the hippocampus of female mice [234], suggesting either the involvement of other ERs (e.g. GPER1) or ER-independent effects of estrogens to influence neurogenesis under chronic conditions.

Much of the work in this area has been conducted in young adult female rodents [218–220, 223–234], but there are a few studies examining middle-age and older females. Curiously, in middle-aged nulliparous females, estrogens lose their ability to increase cell proliferation, but estrone, 17α- and 17β-estradiol all increase cell proliferation in multiparous middle-aged female rats [235]. More work is needed to determine how age and parity affect estrogens’ abilities to modulate neurogenesis.

A few studies conducted in males so far suggest that estradiol does not influence the survival of new neurons, but that both testosterone and dihydrotestosterone increase survival of new neurons [97, 100] *via* interactions with the androgen receptor [236]. The ability of dihydrotestosterone to enhance hippocampal neurogenesis in males depends on age, as the effect is seen in young, but not middle-aged, males [98]. However, Ormerod and colleagues [220] showed that 5 days of exposure to estradiol increased survival of new neurons in male meadow voles, at a time when new neurons are extending their axons in both cage control and voles trained in the Morris Water Maze. This suggests that estradiol can have survival promoting effects in males at specific time points during maturation of new neurons, but more work needs to be done in this area and/or explore species differences. One field that has been neglected is the trajectory of spine formation on new neurons in the dentate gyrus and/or in the CA3 region, the synaptic target of the new neurons. This is a field ripe for investigation.

## Conclusion

The plasticity of the hippocampus that allows for changes and adaptability also makes the hippocampus susceptible to disease and disorder [28]. The literature we review here demonstrates that estrogens can modulate structural plasticity within the hippocampus. A variety of factors, including sex, age, dose, hormonal state, and reproductive history, can influence the effects of estrogens on hippocampal plasticity. Understanding the complex interplay of these factors and estrogens in healthy brains is essential to determining how dysregulation occurs, progresses, and manifests in disease states. A preponderance of research has examined how estrogens affect structural plasticity in female rodents. It is crucial that future studies investigate both sexes, and do so appropriately [1, 2, 237], as many brain disorders show marked sex differences [2]. Additionally, more studies are needed that study estrogens’ effects across age and experience, with the understanding that experiences in one sex may be very different than the other (i.e. motherhood, lactation). Only by understanding the complex, multi-faceted nature of estrogens on hippocampal plasticity can we hope to develop targeted therapies to combat disorders affecting the hippocampus and improve quality of life for those afflicted.

## Abbreviations

4E-BP1: Eukaryotic initiation factor 4E-binding protein
AMPA: Alpha-amino-3-hydroxy-5-methyl-4-isoxazole-propionic acid
BrdU: Bromodeoxyuridine
CaMKII: Ca^2+^/calmodulin-dependent protein kinase II
DPN: Diarylproprionitrile
EB: Estradiol benzoate
ER: Estrogen receptor
ERK: Extracellular signal-regulated protein kinase
GDX: Gonadectomy
GPER1: G-protein-coupled estrogen receptor 1
LTD: Long term depression
LTP: Long term potentiation
mTOR: Mammalian target of rapamycin
NMDA: N-methyl-D-aspartate
OVX: Ovariectomy
PAK: p21-activated kinase
PPT: Propyl pyrazole triol
ROCK: Rho-associated protein kinase

## References

[1] Choleris E, Galea LAM, Sohrabji F, Frick KM (2018). Sex differences in the brain: implications for behavioral and biomedical research. Neurosci Biobehav Rev.

[2] Galea LAM, Frick KM, Hampson E, Sohrabji F, Choleris E (2017). Why estrogens matter for behavior and health. Neurosci Behav Rev.

[3] Angst J, Gamma A, Gastpar M, Lépine JP, Mendlewicz J, Tylee A (2002). Gender differences in depression. Epidemiological findings from the European DEPRES I and II studies. Eur Arch Psychiatry Clin Neurosci.

[4] Barnes LL, Wilson RS, Bienias JL, Schneider JA, Evans DA, Bennett DA (2005). Sex differences in the clinical manifestations of Alzheimer disease pathology. Arch Gen Psychiatry.

[5] Ferri SL, Abel T, Brodkin ES (2018). Sex differences in autism spectrum disorder: a review. Curr Psychiatry Rep.

[6] Irvine K, Laws KR, Gale TM, Kondel TK (2012). Greater cognitive deterioration in women than men with Alzheimer’s disease: a meta analysis. J Clin Exp Neuropsychol.

[7] Mendrek A, Mancini-Marïe A (2016). Sex/gender differences in the brain and cognition in schizophrenia. Neurosci Biobehav Rev.

[8] Gutiérrez-Lobos K, Scherer M, Anderer P, Katschnig H (2002). The influence of age on the female/male ratio of treated incidence rates in depression. BMC Psychiatry.

[9] Kornstein SG, Sloan DM, Thase ME (2002). Gender-specific differences in depression and treatment response. Psychopharmacol Bull.

[10] Kornstein SG, Sloan DM (2003). Gender differences in depression and response to antidepressant treatment. Psychiatr Clin North Am.

[11] Keers R, Aitchison KJ (2010). Gender differences in antidepressant drug response. Int Rev Psychiatry.

[12] Baum LW (2005). Sex, hormones, and Alzheimer’s disease. J Gerontol A Biol Sci Med Sci.

[13] Bloch M, Daly RC, Rubinow DR (2003). Endocrine factors in the etiology of postpartum depression. Compr Psychiatry.

[14] Rosario ER, Chang L, Head EH, Stanczyk FZ, Pike CJ (2011). Brain levels of sex steroid hormones in men and women during normal aging and in Alzheimer’s disease. Neurobiol Aging.

[15] Cui J, Shen Y, Li R (2013). Estrogen synthesis and signaling pathways during aging: from periphery to brain. Trends Mol Med.

[16] Maki PM (2013). The critical window hypothesis of hormone therapy and cognition: a scientific update on clinical studies. Menopause.

[17] Rubinow DR, Johnson SL, Schmidt PJ, Girdler S, Gaynes B (2015). Efficacy of estradiol in perimenopausal depression: so much promise and so few answers. Depress Anxiety.

[18] Lindamer LA, Buse DC, Lohr JB, Jeste DV (2001). Hormone replacement therapy in postmenopausal women with schizophrenia: positive effect on negative symptoms?. Biol Psychiatry.

[19] Hogervorst E, Bandelow S, Moffat SD (2005). Increasing testosterone levels and effects on cognitive functions in elderly men and women: a review. Curr Drug Targets CNS Neurol Disord.

[20] Hogervorst E, Williams J, Budge M, Riedel W, Jolles J (2000). The nature of the effect of female gonadal hormone replacement therapy on cognitive function in post-menopausal women: a meta-analysis. Neuroscience.

[21] Cha J, Greenberg T, Song I, Simpson HB, Posner J, Mujica-Parodi LR (2016). Abnormal hippocampal structure and function in clinical anxiety and comorbid depression. Hippocampus.

[22] Finke C, Bruehl H, Düzel E, Heekeren HR, Ploner CJ (2013). Neural correlates of short-term memory reorganization in humans with hippocampal damage. J Neuroscience.

[23] Cooper RA, Richter FR, Bays PM, Plaisted-Grant KC, Baron-Cohen S, Simons JS (2017). Reduced hippocampal functional connectivity during episodic memory retrieval in autism. Cereb Cortex.

[24] Lieberman JA, Girgis RR, Brucato G, Moore H, Provenzano F, Kegeles L (2018). Hippocampal dysfunction in the pathophysiology of schizophrenia: a selective review and hypothesis for early detection and intervention. Mol Psychiatry.

[25] McKinnon MC, Yucel K, Nazarov A, MacQueen GM (2009). A meta-analysis examining clinical predictors of hippocampal volume in patients with major depressive disorder. J Psychiatry Neurosci.

[26] Sheline YI (2011). Depression and the hippocampus: cause or effect?. Biol Psychiatry.

[27] MacQueen G, Frodl T (2011). The hippocampus in major depression: evidence for the convergence of the bench and bedside in psychiatric research?. Mol Psychiatry.

[28] Leuner B, Gould E (2010). Structural plasticity and hippocampal function. Annu Rev Psychol.

[29] Wharton W, Gleason CE, Sandra O, Carlsson CM, Asthana S (2012). Neurobiological underpinnings of the estrogen-mood relationship. Curr Psychiatry Rev.

[30] Girijala RL, Sohrabji F, Bush RL (2017). Sex differences in stroke: review of current knowledge and evidence. Vasc Med.

[31] Duarte-Guterman P, Yagi S, Chow C, Galea LAM (2015). Hippocampal learning, memory, and neurogenesis: effects of sex, and estrogens across the lifespan in adults. Horm Behav.

[32] Frankfurt M, Luine V (2015). The evolving role of dendritic spines and memory: interaction(s) with estradiol. Horm Behav.

[33] Sheppard PAS, Koss WA, Frick KM, Choleris E (2018). Rapid actions of oestrogens and their receptors on memory acquisition and consolidation in females. J Neuroendocrinol.

[34] Paletta P, Sheppard PAS, Matta R, Ervin KSJ, Choleris E (2018). Rapid effects of estrogens on short-term memory: possible mechanisms. Horm Behav.

[35] Mahmoud R, Wainwright SR, Galea LA (2016). Sex hormones and hippocampal neurogenesis: regulation, implications, and potential mechanisms. Front Neuroendocrinol.

[36] Nugent BM, Tobet SA, Lara HE, Lucion AB, Wilson ME, Recabarren SE (2012). Hormonal programming across the lifespan. Horm Metab Res.

[37] Vannuccini S, Bocchi C, Severi FM, Challis JR, Petraglia F (2016). Endocrinology of human parturition. Ann Endocrinol (Paris).

[38] Windham GC, Elkin E, Fenster L, Waller K, Anderson M, Mitchell PR (2002). Ovarian hormones in premenopausal women: variation by demographic, reproductive and menstrual cycle characteristics. Epidemiology.

[39] Mitra SW, Hoskin E, Yudkovitz J, Pear L, Wilkinson HA, Hayashi S (2003). Immunolocalization of estrogen receptor beta in the mouse brain: comparison with estrogen receptor alpha. Endocrinology.

[40] Hazell GGJ, Yao ST, Roper JA, Prossnitz ER, O’Carroll AM, Lolait SJ (2009). Localisation of GPR30, a novel G protein-coupled oestrogen receptor, suggests multiple functions in the rodent brain and peripheral tissues. J Endocrinol.

[41] Brailoiu E, Dun SL, Brailoiu GC, Mizuo K, Sklar LA, Oprea TI (2007). Distribution and characterization of estrogen receptor G protein-coupled receptor 30 in the rat central nervous system. J Endocrinol.

[42] Nelson LR, Bulun SE (2001). Estrogen production and action. J Am Acad Dermatol.

[43] Burger HG (2002). Androgen production in women. Fertil Steril.

[44] Rannevik G, Jeppsson S, Johnell O, Bjerre B, Laurell-Borulf Y, Svanberg L (1995). A longitudinal study of the perimenopausal transition: altered profiles of steroid and pituitary hormones, SHBG, and bone mineral density. Maturitas.

[45] Budziszewska B, Leskiewicz M, Kubera M, Jaworska-Feil L, Kajta M, Lason W (2001). Estrone, but not 17 beta-estradiol, attenuates kainate-induced seizures and toxicity in male mice. Exp Clin Endocrinol Diabetes.

[46] Bhavnani BR, Berco M, Binkley J (2003). Equine estrogens differentially prevent neuronal cell death induced by glutamate. J Soc Gynecol Investig.

[47] Soldan SS, Alvarez-Retuerto AI, Sicotte NL, Voskuhl RR (2003). Immune modulation in multiple sclerosis patients treated with the pregnancy hormone estriol. J Immunol.

[48] Gatson JW, Liu M-M, Abdelfattah K, Wigginton JG, Smith S, Wolf S (2012). Estrone is neuroprotective in rats after traumatic brain injury. J Neurotrauma.

[49] Ziehn MO, Avedisian AA, Dervin SM, O’Dell TJ, Voskuhl RR (2012). Estriol preserves synaptic transmission in the hippocampus during autoimmune demyelinating disease. Lab Invest.

[50] Falah N, Torday J, Quinney SK, Haas DM (2015). Estriol review: clinical applications and potential biomedical importance. Clin Res Trials.

[51] Gruber CJ, Tschugguel W, Schneeberger C, Huber JC (2002). Production and actions of estrogens. N Engl J Med.

[52] Chen J-R, Yan Y-T, Wang T-J, Chen L-J, Wang Y-J, Tseng G-F (2009). Gonadal hormones modulate the dendritic spine densities of primary cortical pyramidal neurons in adult female rat. Cereb Cortex.

[53] Kinsley CH, Trainer R, Stafisso-Sandoz G, Quadros P, Marcus LK, Hearon C (2006). Motherhood and the hormones of pregnancy modify concentrations of hippocampal neuronal dendritic spines. Horm Behav.

[54] Gould E, Woolley CS, Frankfurt M, McEwen BS (1990). Gonadal steroids regulate dendritic spine density in hippocampal pyramidal cells in adulthood. J Neurosci.

[55] Woolley CS, Gould E, Frankfurt M, McEwen BS (1990). Naturally occurring fluctuation in dendritic spine density on adult hippocampal pyramidal neurons. J Neurosci.

[56] Wallace M, Luine V, Arellano A, Frankfurt M (2006). Ovariectomized rats show decreased recognition memory and spine density in hippocampus and prefrontal cortex. Brain Res.

[57] Luine V, Attalla S, Mohan G, Costa A, Frankfurt M (2006). Dietary phytoestrogens enhance spatial memory and spine density in the hippocampus and prefrontal cortex of ovariectomized rats. Brain Res.

[58] Leranth C, Hajszan T, Szigeti-Buck K, Bober J, MacLusky NJ (2008). Bisphenol A prevents the synaptogenic response to estradiol in hippocampus and prefrontal cortex of ovariectomized nonhuman primates. Proc Natl Acad Sci U.S.A.

[59] Eilam-Stock T, Serrano P, Frankfurt M, Luine V (2012). Bisphenol-A impairs memory and reduces dendritic spine density in adult male rats. Behav Neurosci.

[60] Inagaki T, Frankfurt M, Luine V (2012). Estrogen-induced memory enhancements are blocked by acute bisphenol A in adult female rats: role of dendritic spines. Endocrinology.

[61] Bowman RE, Luine V, Khandaker H, Villafane JJ, Frankfurt M (2014). Adolescent bisphenol-A exposure decreases dendritic spine density: role of sex and age. Synapse.

[62] Bowman RE, Luine V, Weinstein SD, Khandaker H, DeWolf S, Frankfurt M (2015). Bisphenol-A exposure during adolescence leads to enduring alterations in cognition and dendritic spine density in adult male and female rats. Horm Behav.

[63] Dong Y, Jiang A, Yang H, Chen H, Wang Y. Phytoestrogen α-zearalanol improves memory impairment and hippocampal neurogenesis in ovariectomized mice. Sci World J. 2014;2014:862019. 10.1155/2014/862019. 10.1155/2014/862019 PMC4131096 25143992

[64] Harris KM, Kater SB (1994). Dendritic spines: cellular specializations imparting both stability and flexibility to synaptic function. Annu Rev Neurosci.

[65] Alvarez VA, Sabatini BL (2007). Anatomical and physiological plasticity of dendritic spines. Annu Rev Neurosci.

[66] Srivastava DP (2012). Two-step wiring plasticity: a mechanism for estrogen-induced rewiring of cortical neurons. J Steroid Biochem Mol Biol.

[67] Hotulainen P, Hoogenraad CC (2010). Actin in dendritic spines: connecting dynamics to function. J Cell Biol.

[68] Mendell AL, Atwi S, Bailey CD, McCloskey D, Scharfman HE, MacLusky NJ (2017). Expansion of mossy fibers and CA3 apical dendritic length accompanies the fall in dendritic spine density after gonadectomy in male, but not female, rats. Brain Struct Funct.

[69] Shors TJ, Chua C, Falduto J (2001). Sex differences and opposite effects of stress on dendritic spine density in the male versus female hippocampus. J Neurosci.

[70] Rochefort NL, Konnerth A (2012). Dendritic spines: from structure to *in vivo* function. EMBO Rep.

[71] Lee KFH, Soares C, Béïque J-C. Examining form and function of dendritic spines. Neural Plast. 2012;2012:704103. 10.1155/2012/704103. 10.1155/2012/704103 PMC3345238 22577585

[72] Bailey CH, Kandel ER, Harris KM (2015). Structural components of synaptic plasticity and memory consolidation. Cold Spring Harb Perspect Biol.

[73] Bourne J, Harris KM (2007). Do thin spines learn to be mushroom spines that remember?. Curr Op Neurobiol.

[74] Phan A, Lancaster KE, Armstrong JN, MacLusky NJ, Choleris E (2011). Rapid effects of estrogen receptor alpha and beta selective agonists on learning and dendritic spines in female mice. Endocrinology.

[75] Phan A, Gabor CS, Favaro KJ, Kaschack S, Armstrong JN, Maclusky NJ (2012). Low doses of 17β-estradiol rapidly improve learning and increase hippocampal dendritic spines. Neuropsychopharmacology.

[76] Phan A, Suschkov S, Molinaro L, Reynolds K, Lymer JM, Bailey CDC (2015). Rapid increases in immature synapses parallel estrogen-induced hippocampal learning enhancements. Proc Natl Acad Sci U.S.A.

[77] Gabor CS, Lymer J, Phan A, Choleris E (2015). Rapid effects of the G-protein coupled oestrogen receptor (GPER) on learning and dorsal hippocampus dendritic spines in female mice. Physiol Behav.

[78] Tuscher JJ, Luine V, Frankfurt M, Frick KM (2016). Estradiol-mediated spine changes in the dorsal hippocampus and medial prefrontal cortex of ovariectomized female mice depend on ERK and mTOR activation in the dorsal hippocampus. J Neurosci.

[79] Tang Y, Janssen WG, Hao J, Roberts JA, McKay H, Lasley B (2004). Estrogen replacement increases spinophilin-immunoreactive spine number in the prefrontal cortex of female rhesus monkeys. Cereb Cortex.

[80] Khan MM, Dhandapani KM, Zhang Q, Brann DW (2013). Estrogen regulation of spine density and excitatory synapses in rat prefrontal and somatosensory cerebral cortex. Steroids.

[81] Frankfurt M, Gould E, Woolley CS, McEwen BS (1990). Gonadal steroids modify dendritic spine density ventromedial hypothalamic neurons: a Golgi study in the adult rat. Neuroendocrinology.

[82] de Castilhos J, Forti CD, Achaval M, Rasia-Filho AA (2008). Dendritic spine density of posterodorsal medial amygdala neurons can be affected by gonadectomy and sex steroid manipulations in adult rats: a Golgi study. Brain Res.

[83] Rasia-Filho AA, Dalpian F, Menezes IC, Brusco J, Moreira JE, Cohen RS (2012). Dendritic spines of the medial amygdala: plasticity, density, shape, and subcellular modulation by sex steroids. Histol Histopathol.

[84] Woolley CS, McEwen BS (1992). Estradiol mediates fluctuation in hippocampal synapse density during the estrous cycle in the adult rat. J Neurosci.

[85] Woolley CS, McEwen BS (1993). Roles of estradiol and progesterone in regulation of hippocampal dendritic spine density during the estrous cycle in the rat. J Comp Neurol.

[86] Protopopescu X, Butler T, Pan H, Root J, Alternus M, Polanecsky M (2008). Hippocampal structural changes across the menstrual cycle. Hippocampus.

[87] Pletzer B, Kronbichler M, Aichhorn M, Bergmann J, Ladurner G, Kerschbaum MM (2010). Menstrual cycle and hormonal contraceptive use modulate human brain structure. Brain Res.

[88] Lisofsky N, Mårtensson J, Eckert A, Lindenberger U, Gallinat J, Kühn S (2015). Hippocampal volume and functional connectivity changes during the female menstrual cycle. NeuroImage.

[89] Pletzer B, Harris TA, Hidalgo-Lopez E (2018). Subcortical structural changes along the menstrual cycle: beyond the hippocampus. Sci Rep.

[90] Qiu LR, Germann J, Spring S, Alm C, Vousden DA, Palmert MR (2013). Hippocampal volumes differ across the mouse estrous cycle, can change within 24 hours, and associate with cognitive strategies. Neuroimage.

[91] Blume SR, Freedberg M, Vantrease JE, Chan R, Padival M, Record MJ (2017). Sex- and estrus-dependent differences in rat basolateral amygdala. J Neurosci.

[92] Dietrich T, Krings T, Neulen J, Willmes K, Erberich S, Thron A (2001). Effects of blood estrogen level on cortical activation patterns during cognitive activation as measured by functional MRI. Neuroimage.

[93] Zhu X, Kelly TH, Curry TE, Lal C, Joseph JE (2015). Altered functional brain asymmetry for mental rotation: effect of estradiol changes across the menstrual cycle. Neuroreport.

[94] Bayer J, Gläscher J, Finsterbusch J, Schulte LH, Sommer T (2018). Linear and inverted U-shaped dose-response functions describe estrogen effects on hippocampal activity in young women. Nat Commun.

[95] Leranth C, Petnehazy O, MacLusky NJ (2003). Gonadal hormones affect spine synaptic density in the CA1 hippocampal subfield of male rats. J Neurosci.

[96] MacLusky NJ, Hajszan T, Leranth C (2004). Effects of dehydroepiandrosterone and flutamide on hippocampal CA1 spine synapse density in male and female rats: implications for the role of androgens in maintenance of hippocampal structure. Endocrinology.

[97] Spritzer MD, Galea LAM (2007). Testosterone and dihydrotestosterone, but not estradiol, enhance survival of new hippocampal neurons in adult male rats. Devel Neurobiol.

[98] Duarte-Guterman P, Hamson D, Wainwright S, Chow C, Chaiton J, Lieblich SE, et al. Androgens enhance adult hippocampal neurogenesis in males but not females in an age-dependent manner. bioRxiv. 2019;539296. 10.1101/539296. 10.1210/en.2019-00114 PMC6736050 31219567

[99] MacLusky NJ, Hajszan T, Johansen JA, Jordan CL, Leranth C (2006). Androgen effects on hippocampal CA1 spine synapse numbers are retained in *Tfm* male rats with defective androgen receptors. Endocrinology.

[100] Hatanaka Y, Hojo Y, Mukai H, Murakami G, Komatsuzaki Y, Kim J (2015). Rapid increases of spines by dihydrotestosterone and testosterone in hippocampal neurons: dependence on synaptic androgen receptor and kinase networks. Brain Res.

[101] Murakami G, Tsurugizawa T, Hatanaka Y, Komatsuzaki Y, Tanabe N, Mukai H (2006). Comparison between basal and apical dendritic spines in estrogen-induced spinogenesis in CA1 principal neurons in adult hippocampus. Biochem Biophys Res Commun.

[102] Mukai H, Tsurugizawa T, Murakami G, Kominami S, Ishii H, Ogiue-Ikeda M (2007). Rapid modulation of long-term depression and spinogenesis via synaptic estrogen receptors in hippocampal principal neurons. J Neurochem.

[103] Jacome LF, Barateli K, Buitrago D, Lema F, Frankfurt M, Luine VN (2016). Gonadal hormones rapidly enhance spatial memory and increase hippocampal spine density in male rats. Endocrinology.

[104] Kow L-M, Pfaff DW (2016). Rapid estrogen actions on ion channels: a survey in search for mechanisms. Steroids.

[105] Laredo SA, Landeros RV, Trainor BC (2014). Rapid effects of estrogens on behavior: environmental modulation and molecular mechanisms. Front Neuroendocrinol.

[106] Srivastava DP, Woolfrey KM, Penzes P (2013). Insights into rapid modulation of neuroplasticity by brain estrogens. Pharmacol Rev.

[107] MacLusky NJ, Luine VN, Hajszan T, Leranth C (2005). The 17alpha and 17beta isomers of estradiol both induce rapid spine synapse formation in the CA1 hippocampal subfield of ovariectomized female rats. Endocrinology.

[108] Luine VN, Jacome LF, Maclusky NJ (2003). Rapid enhancement of visual and place memory by estrogens in rats. Endocrinology.

[109] Frick KM, Fernandez SM, Bennett JC, Prange-Kiel J, MacLusky NJ, Leranth C (2004). Behavioral training interferes with the ability of gonadal hormones to increase CA1 spine synapse density in ovariectomized female rats. Eur J Neurosci.

[110] Chu X, Gagnidze K, Pfaff D, Ågmo A (2015). Estrogens, androgens and generalized behavioral arousal in gonadectomized female and male C57BL/6 mice. Physiol Behav..

[111] Balthazart J, Choleris E, Remage-Healey L (2018). Steroids and the brain: 50 years of research, conceptual shifts and the ascent of non-classical and membrane-initiated actions. Horm Behav.

[112] Srivastava DP, Woolfrey KM, Jones KA, Shum CY, Lash LL, Swanson GT (2008). Rapid enhancement of two-step wiring plasticity by estrogen and NMDA receptor activity. Proc Natl Acad Sci U.S.A.

[113] Sellers KJ, Erli F, Raval P, Watson IA, Chen D, Srivastava DP (2015). Rapid modulation of synaptogenesis and spinogenesis by 17β-estradiol in primary cortical neurons. Front Cell Neurosci.

[114] Lymer JM, Sheppard PAS, Kuun T, Blackman A, Jani N, Mahbub S (2018). Estrogens and their receptors in the medial amygdala facilitate social recognition in female mice. Psychoneuroendocrinology.

[115] Fan L, Zhao Z, Orr PT, Chambers CH, Lewis MC, Frick KM (2010). Estradiol-induced object memory consolidation in middle-aged female mice requires dorsal hippocampal extracellular signal-regulated kinase and phosphatidylinositol 3-kinase activation. J Neurosci.

[116] Fernandez SM, Lewis MC, Pechenino AS, Harburger LL, Orr PT, Gresack JE (2008). Estradiol-induced enhancement of object memory consolidation involves hippocampal ERK activation and membrane-bound estrogen receptors. J Neurosci.

[117] Fortress AM, Fan L, Orr PT, Zhao Z, Frick KM (2013). Estradiol-induced object recognition memory consolidation is dependent on activation of mTOR signaling in the dorsal hippocampus. Learn Mem.

[118] Holmes MM, Wide JK, Galea LA (2002). Low levels of estradiol facilitate, whereas high levels of estradiol impair, working memory performance on the radial arm maze. Behav Neurosci.

[119] Lewis MC, Kerr KM, Orr PT, Frick KM (2008). Estradiol-induced enhancement of object memory consolidation involves NMDA receptors and protein kinase A in the dorsal hippocampus of female C57BL/6 mice. Behav Neurosci.

[120] Packard MG, Teather LA (1997). Intra-hippocampal estradiol infusion enhances memory in ovariectomized rats. Neuroreport.

[121] Packard MG, Teather LA (1997). Posttraining estradiol injections enhance memory in ovariectomized rats: cholinergic blockade and synergism. Neurobiol Learn Mem.

[122] Zhao ZR, Fan L, Frick KM (2010). Epigenetic alterations regulate estradiol-induced enhancement of memory consolidation. Proc Natl Acad Sci U.S.A.

[123] Stuchlik A (2014). Dynamic learning and memory, synaptic plasticity and neurogenesis: an update. Front Behav Neurosci.

[124] Cowan N (2008). What are the differences between long-term, short-term, and working memory?. Prog Brain Res.

[125] Kandel ER, Dudai Y, Mayford MR (2014). The molecular and systems biology of memory. Cell.

[126] Olton DS, Becker J, Handelmann GE (1979). Hippocampus, space, and memory. Behav Brain Sci.

[127] Baddeley AD, Hitch G (1974). Working memory. Psychol Learn Motiv.

[128] Bizon JL, LaSarge CL, Montgomery KS, McDermott AN, Setlow B, Griffith WH (2009). Spatial reference and working memory across the lifespan of male Fischer 344 rats. Neurobiol Aging.

[129] Moser E, Moser MB, Andersen P (1993). Spatial learning impairment parallels the magnitude of dorsal hippocampal lesions, but is hardly present following ventral lesions. J Neurosci.

[130] Kjelstrup KG, Tuvnes FA, Steffenach H-A, Murison R, Moser EI, Moser M-B (2002). Reduced fear expression after lesions of the ventral hippocampus. Proc Natl Acad Sci U.S.A.

[131] Nott A, Levin ED (2006). Dorsal hippocampal α7 and α4β2 nicotinic receptors and memory. Brain Res.

[132] Yagi S, Chow C, Lieblich SE, Galea LAM (2016). Sex and strategy use matters for pattern separation, adult neurogenesis, and immediate early gene expression in the hippocampus. Hippocampus.

[133] Eichenbaum H, Dudchenko P, Wood E, Shapiro M, Tanila H (1999). The hippocampus, memory, and place cells: is it spatial memory or a memory space?. Neuron.

[134] Bussey TJ, Saksida LM (2007). Memory, perception, and the ventral visual-perirhinal-hippocampal stream: thinking outside of the boxes. Hippocampus.

[135] Bird CM, Burgess N (2008). The hippocampus and memory: insights from spatial processing. Nat Rev Neurosci.

[136] Moscovitch M, Cabeza R, Winocur G, Nadel L (2016). Episodic memory and beyond: the hippocampus and neocortex in transformation. Annu Rev Psychol.

[137] Wide JK, Hanratty K, Ting J, Galea LA (2004). High level estradiol impairs and low level estradiol facilitates non-spatial working memory. Behav Brain Res.

[138] Hampson E (1990). Estrogen-related variations in human spatial and articulatory-motor skills. Psychoneuroendocrinology.

[139] Frye C (1995). Estrus-associated decrements in water maze task are limited to acquisition. Physiol Behav.

[140] Jacome LF, Gautreaux C, Inagaki T, Mohan G, Alves S, Lubbers LS (2010). Estradiol and ERβ agonists enhance recognition memory, and DPN, an ERβ agonist, alters brain monoamines. Neurobiol Learn Mem.

[141] Warren SG, Juraska JM (1997). Spatial and nonspatial learning across the rat estrous cycle. Behav Neurosci.

[142] Holtmaat AJ, Trachtenberg JT, Wilbrecht L, Shepherd GM, Zhang X, Knott GW (2005). Transient and persistent dendritic spines in the neocortex in vivo. Neuron.

[143] Lai K-O, Jordan BA, Ma X-M, Srivastava DP, Tolias KF. Molecular mechanisms of dendritic spine development and plasticity. Neural Plast. 2016;2016:2078121. 10.1155/2016/2078121. 10.1155/2016/2078121 PMC4834162 27127656

[144] Lai K-O, Ip NY (2013). Structural plasticity of dendritic spines: the underlying mechanisms and its dysregulation in brain disorders. Biochim Biophys Acta, Mol Basis Dis.

[145] Dent EW, Merriam EB, Hu X (2011). The dynamic cytoskeleton: backbone of dendritic plasticity. Curr Opin Neurobiol.

[146] Bliss TV, Collingridge GL (1993). A synaptic model of memory: long-term potentiation in the hippocampus. Nature.

[147] Cooke SF, Bliss TV (2006). Plasticity in the human central nervous system. Brain.

[148] Barabás K, Godó S, Lengyel F, Ernszt D, Pál J, Ábrahám IM (2018). Rapid non-classical effects of steroids on the membrane receptor dynamics and downstream signaling in neurons. Horm Behav.

[149] Fernandez SM, Frick KM (2004). Chronic oral estrogen affects memory and neurochemistry in middle-aged female mice. Behav Neurosci.

[150] Galea LA, Wide JK, Paine TA, Holmes MM, Ormerod BK, Floresco SB (2001). High levels of estradiol disrupt conditioned place preference learning, stimulus response learning and reference memory but have limited effects on working memory. Behav Brain Res.

[151] Chesler EJ, Juraska JM (2000). Acute administration of estrogen and progesterone impairs the acquisition of the spatial Morris water maze in ovariectomized rats. Horm Behav.

[152] Gholizadeh S, Sadatmahalleh SJ, Ziaei S (2018). The association between estradiol levels and cognitive function in postmenopausal women. Int J Reprod Biomed (Yazd).

[153] Warren SG, Humphreys AG, Juraska JM, Greenough WT (1995). LTP varies across the estrous cycle: enhanced synaptic plasticity in proestrus rats. Brain Res.

[154] Smejkalova T, Woolley CS (2010). Estradiol acutely potentiates hippocampal excitatory synaptic transmission through a presynaptic mechanism. J Neurosci..

[155] Terasawa E, Timiras PS (1968). Electrical activity during the estrous cycle of the rat: cyclic changes in limbic structures. Endocrinology.

[156] Buterbaugh GG, Hudson GM. Estradiol replacement to female rats facilitates dorsal hippocampal but not ventral hippocampal kindled seizure acquisition. Exp Neurol. 1991;111(1):55-64. 10.1016/0014-4886(91)90050-m 1984433

[157] Wong M, Moss RL (1992). Long-term and short-term electrophysiological effects of on the synaptic properties of hippocampal CA1 neurons. J Neurosci.

[158] Verrotti A, D’Egidio C, Agostinelli S, Verrotti C, Pavone P (2012). Diagnosis and management of catamenial seizures: a review. Int J Womens Health.

[159] Button KS, Ioannidis JPA, Mokrysz C, Nosek BA, Flint J, Robinson ESJ (2013). Power failure: why small sample size undermines the reliability of neuroscience. Nat Rev Neurosci.

[160] Yagi S, Galea LAM (2019). Sex differences in hippocampal cognition and neurogenesis. Neuropsychopharmacology.

[161] Luine VN (2014). Estradiol and cognitive function: past, present and future. Horm Behav.

[162] Inagaki T, Gautreaux C, Luine V (2010). Acute estrogen treatment facilitates recognition memory consolidation and alters monoamine levels in memory-related brain areas. Horm Behav.

[163] Galea LAM, Kavaliers M, Ossenkopp K-P, Hampson E (1995). Gonadal hormone levels and spatial learning performance in Morris water maze in male and female meadow voles, Microtus pennsylvanicus. Horm Behav.

[164] Galea LAM, Ormerod BK, Sampath S, Kostaras X, Wilkie DM, Phelps MT (2000). Spatial working memory and hippocampal size across pregnancy in rats. Horm Behav.

[165] Barha CK, Dalton GL, Galea LA (2010). Low doses of 17α-estradiol and 17β-estradiol facilitate, whereas higher doses of estrone and 17α- and 17β-estradiol impair, contextual fear conditioning in adult female rats. Neuropsychopharmacology.

[166] González-Burgos I, Riviera-Cervantes MC, Velázquez-Zamora DA, Feria-Velasco A, Garcia-Segura LM (2012). Selective estrogen receptor modulators regulate dendritic spine plasticity in the hippocampus of male rats. Neural Plast.

[167] Ervin KS, Mulvale E, Gallagher N, Roussel V, Choleris E (2015). Activation of the G protein-coupled estrogen receptor, but not estrogen receptor α or β, rapidly enhances social learning. Psychoneuroendocrinology.

[168] Gonzales RB, DeLeon Galvan CJ, Rangel YM, Claiborne BJ (2001). Distribution of thorny excrescences on CA3 pyramidal neurons in the rat hippocampus. J Comp Neurol.

[169] Tsurugizawa T, Mukai H, Tanabe N, Murakami G, Hojo Y, Kominami S (2005). Estrogen induces rapid decrease in dendritic thorns in CA3 pyramidal neurons in adult male rat hippocampus. Biochem Biophys Res Commun.

[170] Ethell IM, Pasquale EB (2005). Molecular mechanisms of dendritic spine development and remodeling. Prog Neurobiol.

[171] Halpain S (2000). Actin and the agile spine: how and why do dendritic spines dance?. Trends Neurosci.

[172] Rudy JW (2015). Actin dynamics and the evolution of the memory trace. Brain Res.

[173] Schubert V, Dotti CG (2007). Transmitting on actin: synaptic control of dendritic architecture. J Cell Sci.

[174] Briz V, Baudry M (2014). Estrogen regulates protein synthesis and actin polymerization in hippocampal neurons through different molecular mechanisms. Front Endocrinol (Lausanne).

[175] Flamini MI, Sanchez AM, Goglia L, Tosi V, Genazzani AR, Simoncini T (2009). Differential actions of estrogen and SERMs in regulation of the actin cytoskeleton of endometrial cells. Mol Hum Reprod.

[176] Kramár EA, Chen LY, Brandon NJ, Rex CS, Liu F, Gall CM (2009). Cytoskeletal changes underlie estrogen’s acute effects on synaptic transmission and plasticity. J Neurosci.

[177] Kumar V, Zhang MX, Swank MW, Kunz J, Wu GY (2005). Regulation of dendritic morphogenesis Ras-PI3K-Akt-mTOR and Ras-MAPK signaling pathways. J Neurosci.

[178] Kim J, Schalk JC, Koss WA, Frick KM. The role of actin polymerization in GPER-mediated hippocampal memory enhancement in female mice. Neuroscience 2017 Abstracts. 2017;159.04.

[179] Bramham CR, Wells DG (2007). Dendritic mRNA: transport, translation and function. Nat Rev Neurosci.

[180] Bourtchouladze R, Abel T, Berman N, Gordon R, Lapidus K, Kandel ER (1998). Different training procedures recruit either one or two critical periods for contextual memory consolidation, each of which requires protein synthesis and PKA. Learn Mem.

[181] Schafe GE, Ledoux JE (2000). Memory consolidation of auditory Pavlovian fear conditioning requires protein synthesis and protein kinase A in the amygdala. J Neurosci.

[182] Quevado J, Vianna MRM, Martins MR, Barichello T, Medina JH, Roesler R (2004). Protein synthesis, PKA, and MAP kinase are differentially involved in short- and long-term memory in rats. Behav Brain Res.

[183] Rossato JI, Bevilaqua LRM, Myskiw JC, Medina JH, Izquierdo I, Cammarota M (2007). On the role of hippocampal protein synthesis in the consolidation and reconsolidation of object recognition memory. Learn Mem.

[184] Artinian J, McGauran AM, De Jaeger X, Mouledous L, Frances B, Roullet P (2008). Protein degradation, as with protein synthesis, is required during not only long-term spatial memory consolidation but also reconsolidation. Eur J Neurosci.

[185] Moguel-Gonzalez M, Gomez-Palacio-Schjetnan A, Escobar ML (2008). BDNF reverses the CTA memory deficits produced by inhibition of protein synthesis. Neurobiol Learn Mem.

[186] Kwapis JL, Jarome TJ, Schiff JC, Helmstetter FJ (2011). Memory consolidation in both trace and delay fear conditioning is disrupted by intra-amygdala infusion of the protein synthesis inhibitor anisomycin. Learn Mem.

[187] Sutton MA, Schuman EM (2006). Dendritic protein synthesis, synaptic plasticity, and memory. Cell.

[188] Abraham WC, William JM (2008). LTP maintenance and its protein synthesis-dependence. Neurobiol Learn Mem.

[189] Jarome TJ, Helmstetter FJ (2014). Protein degradation and protein synthesis in long-term memory formation. Front Mol Neurosci.

[190] Noteboom WD, Gorski J (1963). An early effect of estrogen on protein synthesis. Proc Natl Acad Sci U.S.A.

[191] Gorski J, Deangelo AB, Barnea A, McKerns KW (1971). Estrogen action: the role of specific RNA and protein synthesis. The sex steroids: molecular mechanisms.

[192] Nilsson S, Mäkelä S, Treuter E, Tujague M, Thomsen J, Andersson G (2001). Mechanisms of estrogen action. Physiol Rev.

[193] Sarkar SN, Smith LT, Logan SM, Simpkins JW (2010). Estrogen-induced activation of extracellular signal-regulated kinase signaling triggers dendritic resident mRNA translation. Neuroscience.

[194] Sellers K, Raval P, Srivastava DP (2015). Molecular signature of rapid estrogen regulation of synaptic connectivity and cognition. Front Neuroendocrinol.

[195] Waters EM, Mitterling K, Spencer JL, Mazid S, McEwen BS, Milner TA (2009). Estrogen receptor alpha and beta specific agonists regulate expression of synaptic protein in rat hippocampus. Brain Res.

[196] Brake WG, Alves SE, Dunlop JC, Lee SJ, Bulloch K, Allen PB (2001). Novel target sites for estrogen action in the dorsal hippocampus: an examination of synaptic proteins. Endocrinology.

[197] Lee SJ, Romeo RD, Svenningsson P, Campomanes CR, Allen PB, Greengard P (2004). Estradiol affects spinophilin protein differently in gonadectomized males and females. Neuroscience.

[198] Lee SJ, Campomanes CR, Sikat PT, Greenfield AT, Allen PB, McEwen BS (2004). Estrogen induces phosphorylation of cyclic AMP response element binding (pCREB) in primary hippocampal cells in a time-dependent manner. Neuroscience.

[199] Velázquez-Zamora DA, González-Tapia D, González-Ramírez MM, Flores-Soto ME, Vázquez-Valls E, Cervantes M (2012). Plastic changes in dendritic spines of hippocampal CA1 pyramidal neurons from ovariectomized rats after estradiol treatment. Brain Res.

[200] Akama KT, McEwen BS (2003). Estrogen stimulates postsynaptic density-95 synthesis via the Akt/protein kinase B pathway. J Neurosci.

[201] Galea LA (2008). Gonadal hormone modulation of neurogenesis in the dentate gyrus of adult male and female rodents. Brain Res Rev.

[202] van Praag H, Schinder AF, Christie BR, Toni N, Palmer TD, Gage FH (2002). Functional neurogenesis in the adult hippocampus. Nature.

[203] Snyder JS, Kee N, Wojtowicz JM (2001). Effects of adult neurogenesis on synaptic plasticity in the rat dentate gyrus. J Neurophysiol.

[204] Sorrells SF, Paredes MF, Cebrian-Silla A, Sandoval K, Qi D, Kelley KW (2018). Human hippocampal neurogenesis drops sharply in children to undetectable levels in adults. Nature.

[205] Dennis CV, Suh LS, Rodriguez ML, Kril JJ, Sutherland GT (2016). Human adult neurogenesis across the ages: an immunohistochemical study. Neuropathol Appl Neurobiol.

[206] Knoth R, Singec I, Ditter M, Pantazis G, Capetian P, Meyer RP (2010). Murine features of neurogenesis in the human hippocampus across the lifespan from 0 to 100 years. PloS One.

[207] Kornack DR, Rakic P (1999). Continuation of neurogenesis in the hippocampus of the adult macaque monkey. Proc Natl Acad Sci U.S.A.

[208] Eckenhoff MF, Rakic P (1988). Nature and fate of proliferative cells in the hippocampal dentate gyrus during the life span of the rhesus monkey. J Neurosci.

[209] Boldrini M, Fulmore CA, Tartt AN, Simeon LR, Pavlova I, Poposka V (2018). Human hippocampal neurogenesis persists throughout aging. Cell Stem Cell.

[210] Eriksson PS, Perfilieva E, Björk-Eriksson T, Alborn AM, Nordborg C, Peterson DA (1998). Neurogenesis in the adult human hippocampus. Nat Med.

[211] Epp JR, Beasley CL, Galea LAM (2013). Increased hippocampal neurogenesis and p21 expression in depression: dependent on antidepressants, sex, age, and antipsychotic exposure. Neuropsychopharmacology.

[212] Spalding KL, Bergmann O, Alkass K, Bernard S, Salehpour M, Huttner HB (2013). Dynamics of hippocampal neurogenesis in adult humans. Cell.

[213] Kuhn HG, Dickinson-Anson H, Gage FH (1996). Neurogenesis in the dentate gyrus of the adult rat: age-related decrease of neuronal progenitor proliferation. J Neurosci.

[214] Kempermann G, Gage FH, Aigner L, Song H, Curtis MA, Thuret S (2018). Human adult neurogenesis: evidence and remaining questions. Cell Stem Cell.

[215] Tartt AN, Fulmore CA, Liu Y, Rosoklija GB, Dwork AJ, Arango V (2018). Considerations for assessing the extent of hippocampal neurogenesis in the adult and aging human brain. Cell Stem Cell.

[216] Paredes MF, Sorrells SF, Cebrian-Silla A, Sandoval K, Qi D, Kelley KW (2018). Does adult neurogenesis persist in the human hippocampus?. Cell Stem Cell.

[217] Snyder JS (2019). Recalibrating the relevance of adult neurogenesis. Trends Neurosci.

[218] Barha CK, Lieblich SE, Galea LAM (2009). Different forms of oestrogen rapidly upregulate cell proliferation in the dentate gyrus of adult female rats. J Neuroendocrinol.

[219] Ormerod BK, Galea LA (2001). Reproductive status influences cell proliferation and cell survival in the dentate gyrus of adult female meadow voles: a possible regulatory role for estradiol. Neuroscience.

[220] Ormerod BK, Lee TT, Galea LA (2004). Estradiol enhances neurogenesis in the dentate gyri of adult male meadow voles by increasing the survival of young granule neurons. Neuroscience.

[221] Meltzer LA, Yabaluri R, Deisseroth K (2004). A role for circuit homeostasis in adult neurogenesis. Trends Neurosci.

[222] Kuipers SD, Bramham CR, Cameron HA, Fitzsimons CP, Korosi A, Lucassen PJ (2014). Environmental control of adult neurogenesis: from hippocampal homeostasis to behavior and disease. Neural Plast.

[223] Ormerod BK, Lee TT, Galea LA (2003). Estradiol initially enhances but subsequently suppresses (via adrenal steroids) granule cell proliferation in the dentate gyrus of adult female rats. J Neurobiol.

[224] Mazzucco CA, Lieblich SE, Bingham BI, Williamson MA, Viau V, Galea LA (2006). Both estrogen receptor alpha and estrogen receptor beta agonists enhance cell proliferation in the dentate gyrus of adult female rats. Neuroscience.

[225] Ormerod BK, Falconer EM, Galea LA (2003). N-methyl-D-aspartate receptor activity and estradiol: separate regulation of cell proliferation in the dentate gyrus of adult female meadow vole. J Endocrinol.

[226] Duarte-Guterman P, Lieblich SE, Chow C, Galea LA (2015). Estradiol and GPER activation differentially affect cell proliferation but not GPER expression in the hippocampus of adult female rats. PloS ONE.

[227] Tanapat P, Hastings NB, Reeves AJ, Gould E (1999). Estrogen stimulates a transient increase in the number of new neurons in the dentate gyrus of the adult female rat. J Neurosci.

[228] Rummel J, Epp JR, Galea LA (2010). Estradiol does not influence strategy choice but place strategy choice is associated with increased cell proliferation in the hippocampus of female rats. Horm Behav.

[229] Lagace DC, Fischer SJ, Eisch AJ (2007). Gender and endogenous levels of estradiol do not influence adult hippocampal neurogenesis in mice. Hippocampus.

[230] Barha CK, Galea LAM (2013). The hormone therapy, Premarin, impairs hippocampus-dependent spatial learning and memory and reduces activation of new granule neurons in response to memory in female rats. Neurobiol Aging.

[231] McClure RE, Barha CK, Galea LA (2013). 17β-Estradiol, but not estrone, increases the survival and activation of new neurons in the hippocampus in response to spatial memory in adult female rats. Horm Behav.

[232] Barker JM, Galea LA (2008). Repeated estradiol administration alters different aspects of neurogenesis and cell death in the hippocampus of female, but not male, rats. Neuroscience.

[233] Galea LAM, Roes MM, Diemech CJ, Chow C, Mahmoud R, Lieblich SE (2018). Premarin has opposing effects on spatial learning, neural activation, and serum cytokine levels in middle-aged female rats depending on reproductive history. Neurobiol Aging.

[234] Mahmoud R, Duarte-Guterman P, Lieblich SE, Wong SJ, Chaiton JA, Chow C, et al. Neuroplastic and neuroimmune correlates of chronic stress exposure in female mice: modulatory roles of estrogen receptor subtypes. Neuroscience 2018 Abstracts. 2018;234.04.

[235] Barha CK, Galea LA (2011). Motherhood alters the cellular response to estrogens in the hippocampus later in life. Neurobiol Aging.

[236] Hamson DK, Wainwright SR, Taylor JR, Jones BA, Watson NV, Galea LA (2013). Androgens increase survival of adult-born neurons in the dentate gyrus by an androgen receptor-dependent mechanism in male rats. Endocrinology.

[237] Gobinath A, Choleris E, Galea LAM (2017). Sex, hormones, and genotype interact to influence psychiatric disease, treatment and behavioral research. J Neurosci Res.

